# Outcome-guided spike-and-slab Lasso Biclustering: A Novel Approach for Enhancing Biclustering Techniques for Gene Expression Analysis

**DOI:** 10.1007/s11222-025-10709-4

**Published:** 2025-08-28

**Authors:** Luis A. Vargas-Mieles, Paul D. W. Kirk, Chris Wallace

**Affiliations:** 1 Cambridge Institute of Therapeutic Immunology and Infectious Disease (CITIID), University of Cambridge, Cambridge, UK; 2 MRC Biostatistics Unit (BSU), University of Cambridge, Cambridge, UK

**Keywords:** Biclustering, Factor analysis, Profile regression, Spike-and-Slab Lasso

## Abstract

Biclustering has gained interest in gene expression data analysis due to its ability to identify groups of samples that exhibit similar behaviour in specific subsets of genes (or vice versa), in contrast to traditional clustering methods that classify samples based on all genes. Despite advances, biclustering remains a challenging problem, even with cutting-edge methodologies. This paper introduces an extension of the recently proposed Spike-and-Slab Lasso Biclustering (SSLB) algorithm, termed Outcome-Guided SSLB (OG-SSLB), aimed at enhancing the identification of biclusters in gene expression analysis. Our proposed approach integrates disease outcomes into the biclustering framework through Bayesian profile regression. By leveraging additional clinical information, OG-SSLB improves the interpretability and relevance of the resulting biclusters. Comprehensive simulations and numerical experiments demonstrate that OG-SSLB achieves superior performance, with improved accuracy in estimating the number of clusters and higher consensus scores compared to the original SSLB method. Furthermore, OG-SSLB effectively identifies meaningful patterns and associations between gene expression profiles and disease states. These promising results demonstrate the effectiveness of OG-SSLB in advancing biclustering techniques, providing a powerful tool for uncovering biologically relevant insights. The OGSSLB software can be found as an R/C++ package at https://github.com/luisvargasmieles/OGSSLB.

## Introduction

Over the last few decades, the identification of groups that share interesting common characteristics has been a key objective in various real-world applications. Clustering has proven to be a crucial method for discovering these groups that exhibit patterns within high-dimensional data, particularly in the context of large omics datasets (Chauvel et al. 2019). This technique enables the detection of associations between related entities based on shared features or attributes.

One domain of omics in which clustering techniques have been widely employed has been the examination of transcriptomic data, which captures patterns of gene expression levels within biological entities such as tissues or cells (Oyelade et al. 2016; Saelens et al. 2018). The need to understand shared transcriptional patterns embedded in gene expression data has led to extensive development of clustering methodologies.

Although clustering has been beneficial in revealing hidden patterns within these large-scale datasets, it is not without drawbacks. Among its disadvantages, traditional clustering models assume that samples within a cluster behave similarly across all genes and vice versa. Additionally, clustering often results in a partition of the samples or genes into disjoint subsets. These assumptions may oversimplify the biological system under analysis.

Owing to these limitations, biclustering, a methodology that clusters genes and samples simultaneously, has gained more attention in recent years (Xie et al. 2018, 2019; Wang et al. 2021; Gong et al. 2024). This approach allows flexibility in capturing subsets of genes that may behave differently across conditions or subsets of samples that differ according to specific sets of features. Furthermore, biclustering permits overlapping patterns, where genes or samples may belong to multiple biclusters, which more closely matches biological systems. For instance, samples may cluster by sex, disease age and disease state simultaneously, and a single gene may be a member of two or more biological pathways.

Several approaches have been proposed to estimate these subgroups of genes and samples, and various reviews of the biclustering methods developed over the past decades exist in the literature (see, e.g., Eren et al. (2012); Padilha and Campello (2017)). Building upon the results of Nicholls and Wallace (2021), this work focuses on the adoption of a multiplicative model, which has proven advantageous in this context. Such models have demonstrated effective capture of diverse sources of variability in gene expression data, including the presence of outlier genes or genes with fluctuating expression levels (Hochreiter et al. 2010).

Among the existing algorithms using this methodology, we highlight three: Factor analysis for bicluster acquisition (FABIA) (Hochreiter et al. 2010), the BicMix biclustering method (Gao et al. 2016) and Spike-and-Slab Lasso Biclustering (SSLB) (Moran and George 2021). All three are based on a Bayesian factor analysis model with sparsity-inducing priors that have proven to possess a notable ability to recover latent structures in gene expression data. However, a comparative study by Nicholls and Wallace (2021) highlights that SSLB has the advantage of allowing different sparsity levels on each bicluster, in comparison to BicMix, which allows only two levels of sparsity (sparse or dense) for each bicluster, and FABIA, which uses the same sparsity level for all biclusters. Furthermore, while FABIA requires setting the number of biclusters in advance, SSLB (and BicMix) automatically estimates the number of biclusters.

Although recent approaches, such as the ones mentioned above, have been shown to be capable of revealing these latent structures within gene expression data, biclustering, in general, is recognised as an NP-hard problem (Tanay et al. 2002; Peeters 2003). The NP-hardness arises from the challenge of simultaneously grouping rows and columns of a matrix to identify coherent submatrices while considering various constraints and optimisation criteria. Added to the fact that biclusters can also overlap, these difficulties pose a substantial challenge to even the most state-of-the-art methods, further complicating the accurate identification of samples and gene groups that share a common characteristic.

One promising approach to mitigate this complexity is the integration of informative outcome data into the clustering process, thereby guiding the inference towards biologically relevant clustering structures. Several outcome-guided clustering methods have been developed in recent decades, with applications in K-Means clustering (Meng et al. 2022) and gene selection based on survival data (Koestler et al. 2010), to mention a few. For additional insights, see Bair (2013).

In light of these developments, Bayesian profile regression has emerged as another outcome-guided, semi-supervised method for clustering that leverages an outcome variable to inform cluster allocations (Molitor et al. 2010; Liverani et al. 2015). Unlike some of the previously mentioned approaches, it offers a fully model-based framework that can handle a variety of outcome types, making it more versatile. This approach has already shown success in handling binary covariate data (Beall et al. 2024) as well as longitudinal or multivariate continuous outcomes (Rouanet et al. 2023), making it a valuable tool in the context of gene expression analysis.

Building on this success, we explore whether such outcome-guided strategies can also enhance biclustering, where the goal is to simultaneously group genes and samples. Since most gene expression studies also include phenotype information such as age, sex, and disease status, we investigate in this work whether integrating disease outcomes would enhance cluster consistency within specific disease groups. To achieve this, we introduce an outcome-guided version of SSLB by incorporating the disease outcome of the samples into the model via Bayesian profile regression, aiming to better guide the biclustering membership of genes and samples. As we show in numerical and real-data experiments, this integration enhances the accuracy of the SSLB model and refines the biological relevance of the estimated biclusters.

The remainder of the paper is organised as follows. Section 2 discusses the current state of biclustering models, particularly within the context of factor analysis models, and explains the SSLB model that we aim to improve. Section 3 highlights the potential contributions of additional data available in most gene expression studies and presents the methodology used in our work, which integrates Bayesian profile regression into the SSLB model, detailing the computations added to implement this new approach. Section 4 details the results of our experiments and provides a comprehensive analysis of the findings. Finally, Section 5 concludes by summarising the key contributions of our research and suggesting directions for future work.

## Factor Analysis Models and Current Biclustering Techniques

Before presenting the full mathematical formulation, we briefly summarise the modelling approach underlying our work. Our goal is to detect biclusters (groups of genes and patients that co-vary) by leveraging a sparse factor analysis model. In this framework, gene expression data is decomposed into two low-rank matrices capturing patient and gene contributions to each bicluster. We then focus on the Spike-and-Slab Lasso Biclustering (SSLB) method, which uses sparsity-inducing priors to identify interpretable biclusters and forms the basis for the methodological extension we propose in this work.

We assume that gene expression data is represented in a matrix $\textbf{X} \in \mathbb {R}^{N \times G}$ with *N* samples and *G* features or genes. Additionally, we assume that the data $\textbf{X}$ contains *K* non-disjoint latent groups of genes and samples that potentially may be linked due to some common biological characteristics. The problem of determining the number of subgroups *K*, as well as identifying the genes and samples belonging to each of these groups, is defined as “biclustering".

Following the results of Moran and George (2021), we adopt a factor analysis model to identify these latent groups or biclusters, where $\textbf{X}$ can be represented as

1 $$\begin{aligned} \textbf{X} = \mathbf {Z \Lambda ^{\textsf{T}}} + \textbf{E}, \end{aligned}$$

where

- $\textbf{Z} \in \mathbb {R}^{N \times K}$, which will be called the sample loading matrix,
- $\mathbf {\Lambda } \in \mathbb {R}^{G \times K}$, which will be called the gene loading matrix, and
- $\textbf{E} = \left[ \varepsilon _1, \ldots , \varepsilon _N\right] ^{\textsf{T}} \in \mathbb {R}^{N \times G}$ is noise, where each $\varepsilon _i \sim N_G(\textbf{0}, \Sigma )$, and $\Sigma = \textrm{diag} \{\sigma _j^2\}_{j=1}^G$.

Current biclustering algorithms that use the model given in (1) implement an unsupervised approach to identify sparse groups of relevant genes and samples and thus infer $\textbf{Z}$ and $\varvec{\Lambda }$. Their main input is $\textbf{X}$ without additional information (apart from the necessary model parameters) included in the model. For instance, FABIA (Hochreiter et al. 2010) uses a Laplacian prior on all $\textbf{Z}$ and $\varvec{\Lambda }$ entries to induce sparsity, applying the same prior to every entry in these matrices. BicMix (Gao et al. 2016), on the other hand, allows the columns of $\textbf{Z}$ and $\varvec{\Lambda }$ to be sparse or dense. For the sparse components, it utilises three levels of shrinkage, each employing a three-parameter beta (TPB) prior (Armagan et al. 2011), to promote sparsity.

Finally, SSLB employs the Spike-and-Slab Lasso prior (Ročková and George 2018) for both $\textbf{Z}$ and $\varvec{\Lambda }$. This prior enables stronger regularisation on near-zero coefficients (in the spike) to achieve sparsity, while applying weaker regularisation on larger coefficients (in the slab) to maintain accuracy. A key advantage of the SSLB prior over FABIA and BicMix is its ability to allow varying levels of sparsity for each bicluster. As mentioned earlier, FABIA uses the same prior for all biclusters, and BicMix permits only two sparsity levels (‘sparse’ or ‘dense’). SSLB, however, assigns a distinct sparsity parameter to each bicluster.

### The SSLB model

For completeness, we provide a brief explanation of each component within this Bayesian model. See Moran and George (2021) for more details.

#### SSLB likelihood

Since (1) can also be written as

$$\begin{aligned} \textbf{X}=\sum _{k=1}^K \textbf{z}^k \varvec{\lambda }^{k \textsf{T}}+\textbf{E}, \end{aligned}$$

where the superscript $\textbf{z}^k$ represents the *k*th column of $\textbf{Z}$, the likelihood is defined as

$$\begin{aligned} &  p (\textbf{X} \mid \textbf{Z} , \varvec{\Lambda }) \\ &  \quad \propto \prod _{i=1}^N\left\{ \exp \left[ -0.5\left( \textbf{x}_i {-} \textbf{Z}_i \varvec{\lambda }^{\textsf{T}} \right) ^{\textsf{T}} \varvec{\Sigma }^{{-}1}\left( \textbf{x}_i {-} \textbf{Z}_i \varvec{\lambda }^{\textsf{T}} \right) \right] \left( \prod _{j=1}^G \sigma _j^2\right) ^{{-}1 / 2}\right\} , \end{aligned}$$

where the subscript $\textbf{Z}_i$ refers to the *i*th row of $\textbf{Z}$^1^.

#### SSLB priors

*Prior on the elements of* $\varvec{\Lambda }$For the elements of the gene loading matrix, we have a spike-and-slab prior (Ročková and George 2018), defined by

$$\begin{aligned} &  p (\varvec{\Lambda } \mid \varvec{\Gamma }, \omega _0, \omega _1) \propto \prod _{j=1}^G \\ &  \quad \prod _{k=1}^K \left[ \left( 1-\gamma _{j k}\right) \omega _0 \exp \left( -\omega _0\left| \lambda _{j k}\right| \right) \right. \\ &  \quad \left. +\gamma _{j k} \omega _1 \exp \left( -\omega _1\left| \lambda _{j k}\right| \right) \right] , \end{aligned}$$

where $\varvec{\Gamma } = \{ \gamma _{jk} \}_{j, k =1}^{G, K}$ are binary indicator variables that specify if feature *j* is active in bicluster *k*. Depending on $\gamma _{jk}$, each $\lambda _{jk}$ can be drawn from either a Laplacian “spike" characterised by a large parameter value $\omega _0$ and is consequently negligible, or from a Laplacian “slab" with a small parameter $\omega _1$ and, consequently, can be large. Refer to Section 2.1.4 for detailed information on the values of $\omega _0$ and $\omega _1$.

*Prior on the gene binary indicator variable* $\varvec{\Gamma }$o estimate each $\{ \gamma _{jk} \}_{j, k =1}^{G, K}$, the authors use the Beta-Bernoulli prior

$$\begin{aligned} p ( \varvec{\Gamma } \mid \varvec{\Theta }, \alpha ) \propto \prod _{j=1}^G \prod _{k=1}^{K} \theta _k^{\gamma _{j k}+\alpha -1}\left( 1-\theta _k\right) ^{1-\gamma _{j k}}, \end{aligned}$$

where

- $$\varvec{\Theta } = \{\theta _1, \ldots , \theta _K \}$$,
- $$\gamma _{j k} \mid \theta _k \sim \textrm{Bernoulli}(\theta _k)$$,
- $$\theta _k \sim \textrm{Beta}(\alpha , 1)$$.

For this prior, Moran and George (2021) recommends a finite approximation of the Indian buffet process (IBP) prior using $\alpha = 1 / K$. When $K \rightarrow \infty$, this prior is the IBP prior. See Ghahramani and Griffiths (2005) for details.

*Prior on the elements of* $\textbf{Z}$ For the elements of the sample loading matrix, the authors proposed an alternate formulation of the Spike-and-Slab Lasso prior previously defined for the gene loading matrix, for computational purposes. Firstly, an auxiliary variable $\{\tau _{ik}\}_{i,k = 1}^{N, K}$ is introduced in the model, such as

2 $$\begin{aligned} z_{i k} \mid \tau _{i k} \sim N(0, \tau _{i k}), \end{aligned}$$

and then, for each $\tau _{ik}$, a mixture of exponentials is defined as

3 $$\begin{aligned}&p ( \textbf{T} \mid \tilde{\varvec{\Gamma }}, \tilde{\omega }_0, \tilde{\omega }_1) \propto \prod _{i=1}^N \prod _{k=1}^{K} \Bigg [ \left( 1-\tilde{\gamma }_{i k}\right) \tilde{\omega }_0 \exp \left( -0.5 \tilde{\omega }_0 \tau _{i k}\right) \nonumber \\&\quad + \tilde{\gamma }_{i k} \tilde{\omega }_1 \exp \left( -0.5 \tilde{\omega }_1 \tau _{i k}\right) \Bigg ], \end{aligned}$$

where $\tilde{\varvec{\Gamma }}=\left\{ \tilde{\gamma }_{i k}\right\} _{i, k=1}^{N, K}$ are binary indicator variables indicating bicluster membership on the elements of $z_{i k}$, and $\textbf{T}=\left\{ \tau _{i k}\right\} _{i, k=1}^{N, K}$ are the covariances of $z_{i k}$. In summary, the authors represent the Laplace distribution as a scale mixture of a normal with an exponential mixing density: a spike-and-slab Lasso prior on each $z_{i k}$ by introducing auxiliary variables $\tau _{i k}$ for the variance of every $z_{i k}$, and then each $\tau _{i k}$ is assigned a mixture of exponentials (spike-and-slab) priors. Marginalising over the $\tau _{i k}$ yields the usual spike-and-slab Lasso prior.

*Prior on the sample indicator variable* $\tilde{\varvec{\Gamma }}$

For this variable, the authors proposed an Indian Buffet Process (IBP) prior with an optional Pitman-Yor (PY) extension prior (Teh et al. 2007), defined as

4 $$\begin{aligned} \tilde{\gamma }_{i k}&\sim \textrm{Bernoulli}(\tilde{\theta }_{(k)}) \nonumber \\ \tilde{\theta }_{(k)}&= \prod _{l=1}^k \nu _{(l)} \nonumber \\ \nu _{(l)}&\sim \textrm{Beta}(\tilde{\alpha } + ld, 1 - d), \; \; \text {where} \; d \in [0, 1), \; \tilde{\alpha } > - d. \end{aligned}$$

When $0< d < 1$, the above formulation corresponds to the Pitman-Yor IBP prior. In the case where $d = 0$, it represents the standard IBP prior. For the simulations carried out in the SSLB paper and for consistency in this work, the finite approximation to the IBP is also used for comparison, which involves a Beta prior on the sparsity weights, $\tilde{\theta }_k \sim \text {Beta}(\tilde{a}, \tilde{b})$ where $\tilde{a} \propto 1 / K$ and $\tilde{b} = 1$. See Teh et al. (2007) for further details.

*Prior on the covariance matrix* $\varvec{\Sigma }$ *of* $\varepsilon _i$

For the covariance, $\varvec{\Sigma }$, of the vectors $\varepsilon _i$ that define $\textbf{E}$ in (1), an inverse gamma prior was assumed. That is

$$\begin{aligned} p (\varvec{\Sigma } \mid \eta , \xi ) \propto \prod _{j=1}^G\left[ \left( \sigma _j^2\right) ^{-(\eta / 2+1)} \exp \left( \frac{-\eta \xi }{2 \sigma _j^2} \right) \right] , \end{aligned}$$

where the SSLB authors suggest setting $\eta = 3$ and choosing $\xi$ such that the $95\%$ quantile of the prior on $\{\sigma _j^2\}_{j=1}^G$ matches the sample column variance $\{s_j^2\}_{j=1}^G$, i.e., $p(\sigma _j < s_j) = 0.95$. Refer to (Chipman et al. 2010, Section 2.2.4) and (Moran and George 2021, Section 2.5) for further information.

**Fig. 1:**
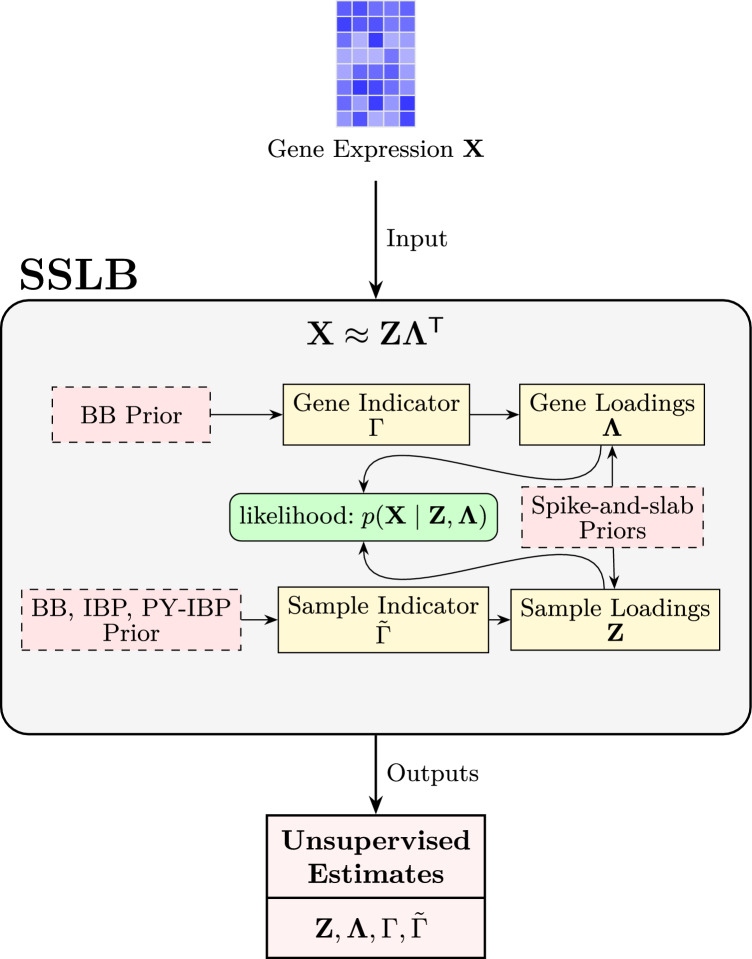
**Overview of SSLB.** The gene expression matrix $\textbf{X}$ is modelled using latent sample and gene loadings, $\textbf{Z}$ and $\mathbf {\Lambda }$, governed by binary indicators $\tilde{\Gamma }$ and $\Gamma$, respectively. Sparsity is induced via spike-and-slab priors, and nonparametric priors are placed on the indicator matrices. The main elements of the model are shown; some components (such as noise variance priors) are omitted for clarity.

**Fig. 2:**
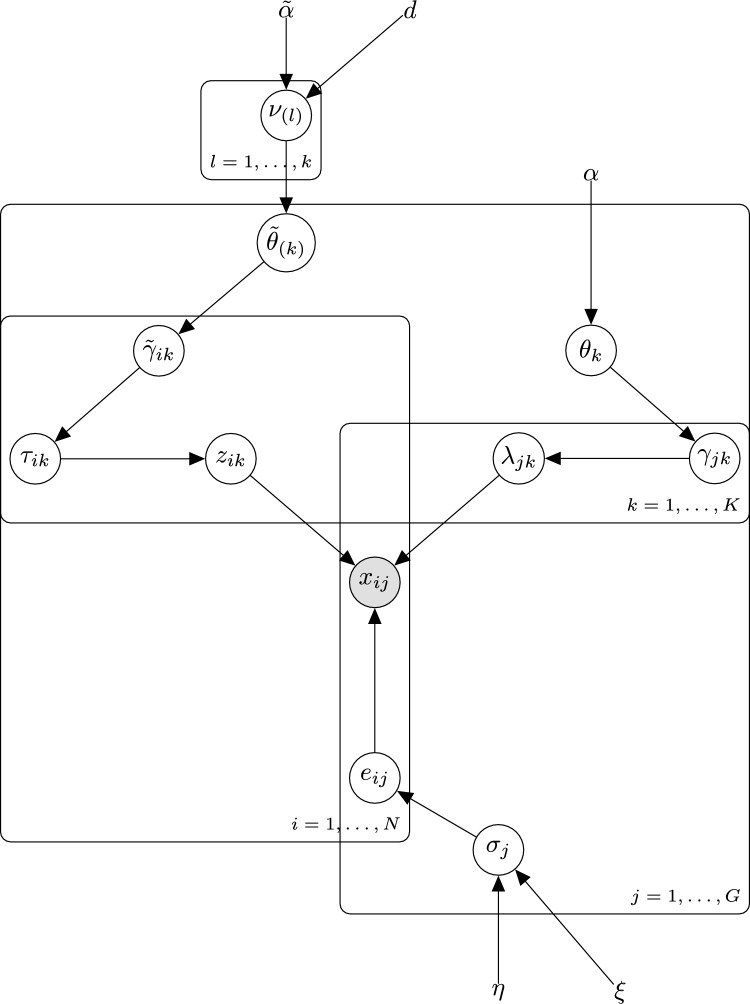
DAG for the SSLB-IBP model, where the indices $i = 1, \dots , N$ correspond to the *N* samples, the indices $j = 1, \dots , G$ correspond to the *G* genes, and the indices $k = 1, \dots , K$ represent the *K* biclusters. Variables $x_{ij}$ are observed and correspond to the gene expression data.

After explaining the whole hierarchical structure of the SSLB model, we first provide a schematic overview of the SSLB model in Figure 1, highlighting its main components and structure. Then, for a more detailed representation of the variable dependencies, we present the corresponding Directed Acyclic Graph (DAG) in Figure 2.

#### Estimation of biclusters: EM algorithm

To proceed with the estimation of the parameters of interest in the SSLB model, the authors implemented an Expectation Maximisation (EM) algorithm. For completeness, we are going to briefly describe the important parts of this procedure for the IBP prior case for $\tilde{\varvec{\Gamma }}$. See (Moran and George 2021, Section 2.3) and its supplementary material for more details.

*E step* At iteration $t+1$ of the EM algorithm, the E step involves the computation of the following expectation

$$\begin{aligned} Q(\varvec{\Delta } \mid \varvec{\Delta }^{(t)})=\mathbb {E}_{\textbf{Z}, \widetilde{\Gamma } \mid \Delta ^{(t)}, \textbf{X}}[\log p (\varvec{\Delta }, \textbf{Z}, \widetilde{\varvec{\Gamma }} \mid \textbf{X})], \end{aligned}$$

where $\varvec{\Delta }=\{\varvec{\Lambda }, \varvec{\Sigma }, \textbf{T}, \nu \}$ are the variables at which $Q(\varvec{\Delta } \mid \varvec{\Delta }^{(t)})$ will be maximised in the M step. See (A1) and (A2) for more details.

*M step* In this stage, the following is calculated

$$\begin{aligned} \varvec{\Delta }^{(t + 1)} = \mathop {\mathrm {arg\,max}}\limits _{\Delta } Q(\varvec{\Delta } \mid \varvec{\Delta }^{(t)}). \end{aligned}$$

#### Implementation of SSLB & initial conditions

The SSLB algorithm employs the previously described EM algorithm combined with a dynamic posterior exploration approach to estimate $\varvec{\Lambda }$. This involves a gradual increase of the spike parameter $\omega _0$ through a sequence of values, while the slab parameter $\omega _1$ is kept fixed. This strategy helps stabilise large coefficients and progressively thresholds negligible coefficients to zero (see (Moran and George 2021, Section 2.3) for more details).

The SSLB algorithm is initialised with entries of $\varvec{\Lambda }$ generated independently from a standard normal distribution. The entries of $\textbf{T}$, the matrix of auxiliary variance parameters, are set to 100, representing an initial relatively non-informative prior to $\textbf{Z}$. The sparsity weights, $\theta _k$, are initialised at 0.5. The IBP parameters, $\varvec{\nu }$, are generated independently from a $\textrm{Beta}(1, 1)$ distribution and then ordered from largest to smallest. In real-world applications, the recommended initialisation for K, the number of biclusters, is set to $K_{init} = 50$. See (Moran and George 2021, Section 2.5) for more details.

## Methodology

Considering the potential availability of additional data on sampled individuals, such as disease status, our objective is to investigate whether incorporating this information can enhance the complex task of biclustering. Notably, to the best of our knowledge, no existing biclustering method has explored the inclusion of the disease status of samples within the model.

We propose incorporating an outcome variable $\textbf{Y} \in \{0, 1\}^{N \times C}$ into the current SSLB model, which will correspond to the presence or absence (i.e., 1 or 0, respectively) of disease $c \in \{1, \ldots , C\}$ in the sample $i \in \{1, \ldots , N\}$.

We assume we have gene expression data $\textbf{X}$ also modelled as (1), and outcomes $\textbf{Y}$. Since $\textbf{Y}$ provides only sample-wise information, it will only affect the distribution related to the sample loading matrix $\textbf{Z}$. The general model considered in Molitor et al. (2010) and adapted for the biclustering problem with respect to $\varvec{\Lambda }$ is defined as:

$$\begin{aligned} p \left( \textbf{Z} , \textbf{Y} \mid \varvec{\Theta }_{\textbf{Z}} , \varvec{\Theta }_{\textbf{Y}} \right) = \prod _{i=1}^N p (\textbf{y}_i \mid \varvec{\theta }_{\textbf{y}_i}, \textbf{z}_i) p (\mathbf {z_i} \mid \varvec{\theta }_{\textbf{z}_i} ) , \end{aligned}$$

where $\varvec{\theta }_{\textbf{z}_i}$ represents the parameters of the model for $\textbf{z}_i$, and $\varvec{\theta }_{\textbf{y}_i}$ represents the parameters of the model for $\textbf{y}_i$. Furthermore, in the profile regression setting, the factor loadings $\textbf{Z}$ and the outcome $\textbf{Y}$ are conditionally independent because their relationship is mediated by the binary indicator matrix $\tilde{\varvec{\Gamma }}$, which governs the structure of the sampling clustering. $\tilde{\varvec{\Gamma }}$ determines which latent factors contribute to $\textbf{Z}$ and how they align with $\textbf{Y}$. Therefore, the profile regression model becomes:

$$\begin{aligned} p \left( \textbf{Z} , \textbf{Y} \mid \varvec{\Theta }_{\textbf{Z}} , \varvec{\Theta }_{\textbf{Y}} \right) = \prod _{i=1}^N p (\textbf{y}_i \mid \varvec{\theta }_{\textbf{y}_i}) p (\mathbf {z_i} \mid \varvec{\theta }_{\textbf{z}_i} ) , \end{aligned}$$

where

- $$\varvec{\Theta }_{\textbf{Z}} = \{ \tilde{\varvec{\Gamma }}, \textbf{T}, \nu , \widetilde{\omega _0}, \widetilde{\omega _1}, \tilde{\varvec{\theta }}, \tilde{\alpha } \}$$.
- $$p \left( \textbf{Z} \mid \varvec{\Theta }_{\textbf{Z}}\right) \propto p ( \textbf{Z} \mid \textbf{T}) p ( \textbf{T} \mid \tilde{\varvec{\Gamma }}, \widetilde{\omega _0}, \widetilde{\omega _1}) p({\tilde{\varvec{\Gamma }}} \mid \tilde{\varvec{\theta }}, \tilde{\alpha })$$.

and $\varvec{\Theta }_{\textbf{Y}}$ is the set of bicluster-specific parameters of the model for $\textbf{Y}$, which also includes $\tilde{\varvec{\Gamma }}$. Note that each of the probability density functions given in $p \left( \textbf{Z} \mid \varvec{\Theta }_{\textbf{Z}}\right)$ is already defined for the current SSLB model in (2), (3), and (4).

To model the disease outcome $\textbf{Y}$, we adopt a multinomial logistic regression approach, where the latent bicluster memberships $\widetilde{\gamma }_{ik}$ serve as covariates. The model can be interpreted through the log-odds of a disease $c \in \{1, \ldots , C-1\}$ relative to a reference disease category $C$, given by:

$$\begin{aligned} \log \frac{P(y_{ic} = 1)}{P(y_{iC} = 1)} = \sum _{k=1}^K w_{ck} \widetilde{\gamma }_{ik}, \end{aligned}$$

where $w_{ck}$ denotes the contribution of bicluster $k$ to the log-odds of disease $c$. This formulation makes it explicit that if sample $i$ belongs to bicluster $k$, it contributes additively to the risk of disease $c$. Unlike standard Bayesian profile regression, our model allows overlapping bicluster membership–that is, each sample can belong to multiple biclusters–providing greater flexibility in capturing complex disease-related structures in gene expression data.

We formalise this log-odds formulation in the full likelihood expression as:

5 $$\begin{aligned} &  p \left( \textbf{Y} \mid \varvec{\Theta }_{\textbf{Y}} \right) \propto \prod _{i=1}^N \prod _{l=1}^C \nonumber \\ &  \quad \left[ \exp \left( \textbf{w}^{(l)\,T} \tilde{\varvec{\gamma }}_i^{\prime }\right) / \sum _{l^{\prime }=1}^c \exp \left( \textbf{w}^{(l^{\prime })\,T} \tilde{\varvec{\gamma }}_i^{\prime }\right) \right] ^{y_{i l}} \nonumber \\ &  \quad + \exp \left( - \frac{1}{2} \zeta _{\text {w}} \Vert \textbf{W} \Vert _{F}^2 \right) , \end{aligned}$$

where

- $$\varvec{\Theta }_{\textbf{Y}} = \{ \tilde{\varvec{\Gamma }}^{\prime }, \textbf{W}, \zeta _{\text {w}} \}$$.
- $y_{i l}$ corresponds to the presence/absence (i.e., 1 or 0) of the *l* disease in the *i* sample.
- *C* is the number of diseases (i.e., the number of categories in the multinomial logistic regression model) in the study.
- $\textbf{W} \in \mathbb {R}^{(K + 1) \times C}$ is the matrix of weights of the multinomial logistic regression, which includes the bias coefficients. It is the main element that allows $\textbf{Y}$ to assist in the task of assigning samples (and genes) to biclusters.
- $$\tilde{\varvec{\gamma }}_i^{\prime } = \left[ 1, \tilde{\gamma }_{i1}, \ldots , \tilde{\gamma }_{iK} \right] $$.
- A reference category (e.g., class or disease *C*) needs to be chosen such that the column of the matrix $\textbf{W}$ corresponding to the selected reference category has only zeros (e.g., $\textbf{w}_C = \left[ 0, \ldots , 0\right]$ ).
- To avoid overfitting, we have introduced an $\ell _2$ regularisation term for the weight matrix $\textbf{W}$ with regularisation hyperparameter $\zeta _{\text {w}} \in \mathbb {R}^+$.

**Fig. 3:**
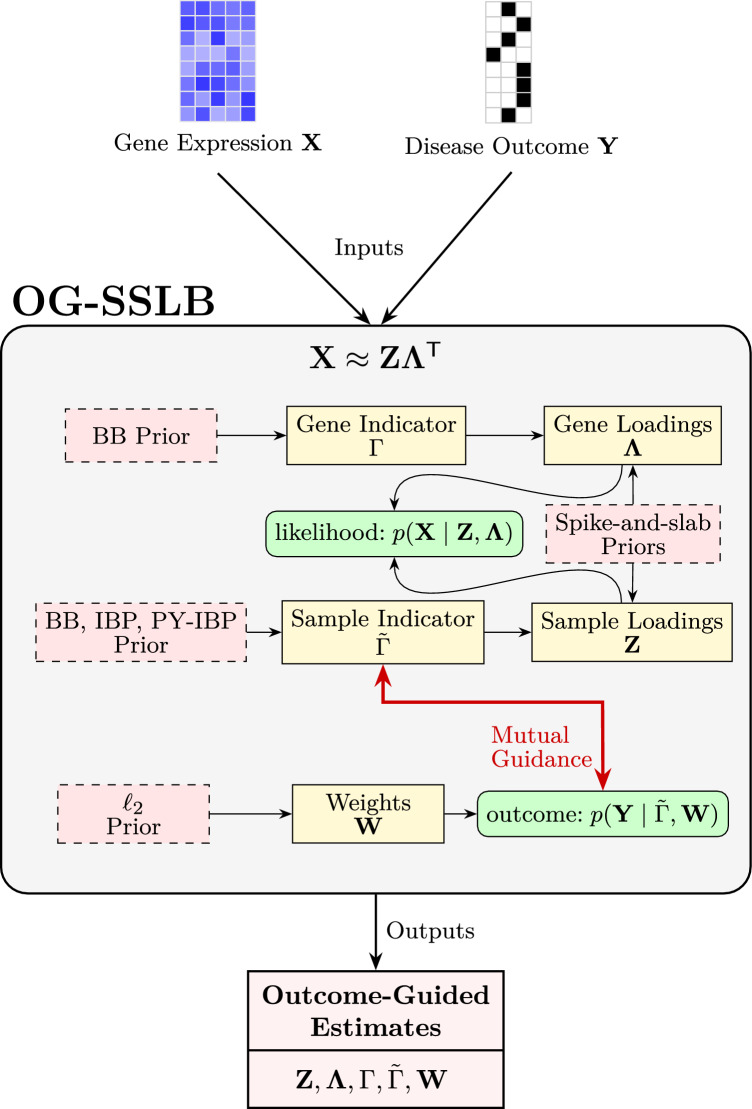
**Overview of OG-SSLB.** The model extends SSLB by incorporating an outcome variable $\textbf{Y}$, linked to the latent structure via the sample indicator matrix $\tilde{\Gamma }$ and regression weights $\textbf{W}$. The outcome model $p(\textbf{Y} \mid \tilde{\Gamma }, \textbf{W})$ plays a dual role: it uses $\tilde{\Gamma }$ as input and, simultaneously, guides its inference. The red bidirectional arrow illustrates this mutual interaction. For clarity, auxiliary components such as noise priors are omitted.

By incorporating this approach into the model, the complete log posterior (A1) and the expression associated with the E step (A2) will undergo minor modifications. A schematic representation of the OG-SSLB model structure, including its outcome-guided component, is shown in Figure 3. See also (B3) and (B4) in the Appendix for details.

### Adapted EM algorithm for OG-SSLB

To estimate the parameters of the OG-SSLB model, we adapt the Expectation-Maximisation (EM) procedure originally proposed for SSLB. The main difference lies in the incorporation of the disease outcome variable $\textbf{Y}$, and the regression parameters $\textbf{W}$ and $\zeta _{\text {w}}$. Below we summarise the updated steps.

*E step* At iteration $t + 1$, the E step involves computing:

$$\begin{aligned} Q(\varvec{\Delta } \mid \varvec{\Delta }^{(t)}) = \mathbb {E}_{\textbf{Z}, \widetilde{\varvec{\Gamma }} \mid \varvec{\Delta }^{(t)}, \textbf{X}, \textbf{Y}} \left[ \log p(\varvec{\Delta }, \textbf{Z}, \widetilde{\varvec{\Gamma }} \mid \textbf{X}, \textbf{Y}) \right] , \end{aligned}$$

where $\varvec{\Delta } = \{\varvec{\Lambda }, \varvec{\Sigma }, \textbf{T}, \nu , \textbf{W}, \zeta _{\text {w}} \}$. Compared to SSLB, this step now also includes:

- estimation of the expectations $\left\langle \widetilde{\gamma }_{ik} \right\rangle$ using the joint information from $\textbf{X}$ and $\textbf{Y}$,
- estimation of the expectation of the log-sum-exp term from the multinomial logistic regression likelihood.

*M step* In the M step, we maximise:

$$\begin{aligned} \varvec{\Delta }^{(t + 1)} = \mathop {\mathrm {arg\,max}}\limits _{\varvec{\Delta }} Q(\varvec{\Delta } \mid \varvec{\Delta }^{(t)}), \end{aligned}$$

which includes updating the standard SSLB parameters $\{\varvec{\Lambda }, \varvec{\Sigma }, \textbf{T}, \nu \}$ and additionally estimating $\textbf{W}$ and $\zeta _{\text {w}}$ via a regularised multinomial logistic regression.

Below we will explain the computation of the new expectation and maximisation steps introduced by the profile regression model adapted to the SSLB model, particularly the computation of $\left\langle \widetilde{\varvec{\gamma }}_i^{\prime }\right\rangle$, the second last term of (B4) that involves the computation of an expectation of a log-sum-exp expression, the estimation of $\zeta _{\text {w}}$ and the maximisation of $\textbf{W}$. See (Moran and George 2021, Section 2.3) and its supplementary material for details on the computation of the remaining parameters that are not affected by the introduction of profile regression to the SSLB model.

### Expectation of $\tilde{\varvec{\gamma }}_i$

For the expectation of the $\widetilde{\gamma }_{i k}$ variables (i.e., the binary membership indicator of sample *i* in bicluster *k*), we have

6 $$\begin{aligned} \left\langle \widetilde{\gamma }_{ik}\right\rangle&=p\left( \widetilde{\gamma }_{ik}=1 \mid \textbf{Y}, \textbf{T}, \widetilde{\varvec{\theta }}, \textbf{W}\right) \nonumber \\ &=\frac{1}{1 + \frac{p\left( y_{ic} \mid \widetilde{\gamma }_{ik}=0, \textbf{w}^c\right) p\left( \tau _{ik} \mid \widetilde{\gamma }_{ik}=0\right) p\left( \widetilde{\gamma }_{ik}=0 \mid \widetilde{\theta }_k\right) }{p\left( y_{ic} \mid \widetilde{\gamma }_{ik}=1, \textbf{w}^c\right) p\left( \tau _{ik} \mid \widetilde{\gamma }_{ik}=1\right) p\left( \widetilde{\gamma }_{ik}=1 \mid \widetilde{\theta }_k\right) }} \end{aligned}$$

where *c* corresponds to the *c*-th disease presented in sample *i*, and $\textbf{w}^c$ is the *c*-th column of the matrix of weights $\textbf{W} \in \mathbb {R}^{(K + 1) \times C}$.

From this, the only new expression left to compute is $p\left( y_{ic} \mid \widetilde{\gamma }_{ik}=1, \textbf{w}^c\right)$, given by

$$\begin{aligned} &  p(y_{i c} \mid \widetilde{\gamma }_{i k}=1, \textbf{w}^c) =\sum _{\widetilde{\gamma }_{i, \backslash k}} p(y_{i c} \mid \widetilde{\gamma }_{i k}=1, \widetilde{\gamma }_{i, \backslash k},\\ &  \quad \textbf{w}^c) p(\widetilde{\gamma }_{i, \backslash k} \mid \widetilde{\gamma }_{i k}=1, \varvec{T}, \widetilde{\varvec{\theta }}). \end{aligned}$$

Since this is not available in closed form, we approximate it using Monte Carlo. Specifically, we

- Generate *M* samples from $p\left( \widetilde{\gamma }_{ik} \mid \textbf{T}, \widetilde{\varvec{\theta }}\right)$, a probability to which we have access (see the supplementary material of Moran and George (2021)). This is done for every $k \in \{1, \ldots , K\}$. The results will be stored in a matrix $\textbf{V} \in \mathbb {R}^{M\times K}$.
- For each column $k \in \{1, \ldots , K\}$ in $\textbf{V}$:
  1. Extract samples of $\varvec{\tilde{\gamma }}$ where $\widetilde{\gamma }_{ik} = 1$; that is, extract only the rows of $\textbf{V}$ whose *k*-th column is equal to 1. Note that these are samples from $p(\widetilde{\gamma }_{i, \backslash k} \mid \widetilde{\gamma }_{i k}=1, \varvec{T}, \widetilde{\varvec{\theta }})$. This subset of $\textbf{V}$ can be defined as $\textbf{V}^{\prime } \in \mathbb {R}^{M^{\prime } \times K}$ where $M^{\prime } \le M$.
  2. Compute $p(y_{i c} \mid \widetilde{\gamma }_{i k}^{(m)}=1, \widetilde{\gamma }_{i, \backslash k}^{m}, \textbf{w}^c)$ using (5) for the samples $m = 1, \ldots , M^{\prime }$.
  3. Finally, estimate $p\left( y_{ic} \mid \widetilde{\gamma }_{ik}=1, \textbf{w}^c\right)$ as
    $$\begin{aligned} p\left( y_{ic} \mid \widetilde{\gamma }_{ik}=1, \textbf{w}^c\right) &  \approx \frac{1}{M^{\prime }} \sum _{m = 1}^{M^{\prime }} p(y_{i c} \mid \widetilde{\gamma }_{i k}^{(m)}=1, \\  &  \quad \widetilde{\gamma }_{i, \backslash k}^{m}, \textbf{w}^c). \end{aligned}$$

In our numerical experiments, we have empirically observed that using $M=50$ results in estimates of $\left\langle \widetilde{\gamma }_{ik}\right\rangle$ with sufficiently low variance and consistent results across multiple trials, indicating that this choice of *M* is sufficient for reliable estimation.

*Connection to mean-field variational inference.* The steps involved in the computation of $\left\langle \widetilde{\gamma }_{ik}\right\rangle$ in our model assume conditional independence across the binary latent indicators $\widetilde{\gamma }_{ik}$, thus approximating the full posterior $p(\widetilde{\varvec{\Gamma }} \mid \textbf{X}, \textbf{Y}, \varvec{\Delta }^{(t)})$ by the product of marginals:

$$\begin{aligned} p(\widetilde{\varvec{\Gamma }} \mid \textbf{X}, \textbf{Y}, \varvec{\Delta }^{(t)}) \approx \prod _{i=1}^N \prod _{k=1}^{K} p(\widetilde{\gamma }_{ik} \mid \textbf{X}, \textbf{Y}, \varvec{\Delta }^{(t)}). \end{aligned}$$

This corresponds to a standard *mean-field variational inference* (MFVI) approximation, where the variational posterior factorises as $q(\widetilde{\varvec{\Gamma }}) = \prod _{i,k} q(\widetilde{\gamma }_{ik})$, with each $q(\widetilde{\gamma }_{ik})$ taken to be a Bernoulli distribution parameterised by the current estimate of $\mathbb {E}[\widetilde{\gamma }_{ik}]$. Under this framework, the optimal update for $q(\widetilde{\gamma }_{ik})$ is given by Equation (6) (Blei et al. 2017).

In addition, the conditional likelihood $p(y_{ic} \mid \widetilde{\gamma }_{ik}=1, \textbf{w}^c)$ depends formally on all other latent indicators $\widetilde{\gamma }_{i,\backslash k}$, that is

$$\begin{aligned} p(y_{ic} \mid \widetilde{\gamma }_{ik}=1, \textbf{w}^c)= &  \sum _{\widetilde{\gamma }_{i, \backslash k}} p(y_{ic} \mid \widetilde{\gamma }_{ik}=1, \widetilde{\gamma }_{i, \backslash k},\\ &  \quad \textbf{w}^c) p(\widetilde{\gamma }_{i, \backslash k} \mid \widetilde{\gamma }_{ik}=1, \textbf{T}, \widetilde{\varvec{\theta }}). \end{aligned}$$

While our Monte Carlo estimate does not explicitly model full dependence among all $\widetilde{\gamma }_{i}$, it is aligned with the mean-field variational approximation assumed. Empirically, this approach yields stable estimates and effective convergence in our experiments. For similar treatments under mean-field assumptions, see also Ročková and George (2014); Carbonetto and Stephens (2012).

### Expectation of the log-sum-exp expression in $Q(\varvec{\Delta } \mid \varvec{\Delta }^{(t)})$

For the computation of the last term of (B4) which implies an expectation of a log-sum-exp expression, since we now have a way to estimate $\left\langle \widetilde{\gamma }_{ik}\right\rangle$, the computation of this expectation is a simple Monte Carlo estimate as follows:

- Generate $\widetilde{\varvec{\gamma }}_i^{\prime (1)}, \ldots , \widetilde{\varvec{\gamma }}_i^{\prime (m)}$ samples from $p\left( \widetilde{\gamma }_{ik}=1 \mid \right.$ $\left. \textbf{Y}, \textbf{T}, \widetilde{\varvec{\theta }}, \textbf{W}\right)$ previously estimated in Section 3.2, for each $k \in \{1, \ldots , K \}$.
- Compute
  $$\begin{aligned} &  \Biggl \langle \log \left[ \sum _{l=1}^C \exp \left( \textbf{w}^{(l)\,T} \widetilde{\varvec{\gamma }}_i^{\prime }\right) \right] \Biggr \rangle \approx \frac{1}{m}\\  &  \quad \sum _{i=1}^{m} \log \left[ \sum _{l=1}^C \exp \left( \textbf{w}^{(l)\,T} \widetilde{\varvec{\gamma }}_i^{\prime (m)} \right) \right] \end{aligned}$$

### Estimation of hyperparameter $\zeta _{\text {w}}$

To estimate the regularisation hyperparameter $\zeta _{\text {w}}$ of the $\ell _2$ penalisation term of the multinomial logistic regression weights matrix $\textbf{W}$, we adopted an empirical Bayesian approach by maximum marginal likelihood estimate. This can be done by solving the following

7 $$\begin{aligned} \zeta _{\text {w}}^{\text{* }}&= \mathop {\mathrm {arg\,max}}\limits _{\zeta _{\text {w}}} p \left( \textbf{Y} \mid \tilde{\varvec{\Gamma }}^{\prime }, \zeta _{\text {w}} \right) \nonumber \\&= \mathop {\mathrm {arg\,max}}\limits _{\zeta _{\text {w}}} \int _{\mathbb {R}^{(K + 1) \times C}} \prod _{i=1}^N \prod _{l=1}^C\left[ \exp \left( \textbf{w}^{(l)\,T}\tilde{\varvec{\gamma }}_i^{\prime }\right) / \sum _{l^{\prime }=1}^c \nonumber \right. \\&\quad \left. \exp \left( \textbf{w}^{(l^{\prime })\,T} \tilde{\varvec{\gamma }}_i^{\prime }\right) \right] ^{y_{i l}} \nonumber \\&\quad + \exp \left( - \frac{1}{2} \zeta _{\text {w}} \Vert \textbf{W} \Vert _{F}^2 \right) d \textbf{W} . \end{aligned}$$

Since the latter integral, i.e., the resulting marginal likelihood of the multinomial logistic regression model, is computationally intractable, we will apply the Stochastic Optimisation via Unadjusted Langevin (SOUL) method (De Bortoli et al. 2021), which is specifically designed for this type of problem. We will explain this method in detail below.

We can solve (7) iteratively using the projected gradient algorithm (Levitin and Polyak 1966, Section 5)

8 $$\begin{aligned} \zeta _{\text {w}}^{(n+1)} = \Pi _{\Theta _{\zeta _{\text {w}}}} \left[ \zeta _{\text {w}}^{(n)} + \delta _{\text {PGA}}^{(n)} \nabla _{\zeta _{\text {w}}} p \left( \textbf{Y} \mid \tilde{\varvec{\Gamma }}^{\prime }, \zeta _{\text {w}}^{(n)} \right) \right] , \end{aligned}$$

by computing a sequence $(\zeta _{\text {w}}^{(n)})_{n \in \mathbb {N}}$ associated with the latter recursion, where $\Pi _{\Theta _{\zeta _{\text {w}}}}$ denotes the projection onto the compact convex set $\Theta _{\zeta _{\text {w}}} \subset (0,+\infty )$ and $(\delta _{\text {PGA}}^{(n)})_{n \in \mathbb {N}}$ is a sequence of non-increasing step sizes^2^. However, the gradient in (8) is intractable, as we saw in (7). For this case, we can replace this gradient with a stochastic estimator by applying Fisher’s identity (Douc et al. 2014, Section D.2)

$$\begin{aligned}&\nabla _{\zeta _{\text {w}}} p \left( \textbf{Y} \mid \tilde{\varvec{\Gamma }}^{\prime }, \zeta _{\text {w}} \right) \\&\quad {=} \int _{\mathbb {R}^{(K {+} 1) {\times } C}} \frac{\nabla _{\zeta _{\text {w}}} p \left( \textbf{W}, \textbf{Y} \mid \tilde{\varvec{\Gamma }}^{\prime }, \zeta _{\text {w}} \right) }{p \left( \textbf{W}, \textbf{Y} \mid \tilde{\varvec{\Gamma }}^{\prime }, \zeta _{\text {w}} \right) } p \left( \textbf{W} \mid \textbf{Y}, \tilde{\varvec{\Gamma }}^{\prime }, \zeta _{\text {w}} \right) d \textbf{W} \\&\quad = \int _{\mathbb {R}^{(K + 1) \times C}} \nabla _{\zeta _{\text {w}}} \\&\quad \log p \left( \textbf{W}, \textbf{Y} \mid \tilde{\varvec{\Gamma }}^{\prime }, \zeta _{\text {w}} \right) p \left( \textbf{W} \mid \textbf{Y}, \tilde{\varvec{\Gamma }}^{\prime }, \zeta _{\text {w}} \right) d \textbf{W}, \end{aligned}$$

5 where $p (\textbf{W} \mid \textbf{Y}, \tilde{\varvec{\Gamma }}^{\prime }, \zeta _{\text {w}} )$ is the posterior distribution of $\textbf{W}$, given by

$$\begin{aligned} p \left( \textbf{W} \mid \textbf{Y}, \tilde{\varvec{\Gamma }}^{\prime }, \zeta _{\text {w}} \right) \propto p \left( \textbf{Y} \mid \textbf{W}, \tilde{\varvec{\Gamma }}^{\prime }, \zeta _{\text {w}} \right) p \left( \textbf{W} \mid \zeta _{\text {w}} \right) . \end{aligned}$$

Given the fact that $p (\textbf{W}, \textbf{Y} \mid \tilde{\varvec{\Gamma }}^{\prime }, \zeta _{\text {w}} ) = p (\textbf{Y} \mid \textbf{W}, \tilde{\varvec{\Gamma }}^{\prime }) p (\textbf{W} \mid \zeta _{\text {w}})$ we have

$$\begin{aligned} \nabla _{\zeta _{\text {w}}} p \left( \textbf{Y} \mid \tilde{\varvec{\Gamma }}^{\prime }, \zeta _{\text {w}} \right)&= \int _{\mathbb {R}^{(K + 1) \times C}} \nabla _{\zeta _{\text {w}}} \log p \left( \textbf{W} \mid \zeta _{\text {w}} \right) p \left( \textbf{W} \mid \right. \\&\quad \left. \textbf{Y},\tilde{\varvec{\Gamma }}^{\prime }, \zeta _{\text {w}} \right) d \textbf{W} \end{aligned}$$

where

$$\begin{aligned} \log p \left( \textbf{W} \mid \zeta _{\text {w}} \right)&= - \frac{1}{2} \zeta _{\text {w}} \Vert \textbf{W} \Vert _{F}^2 - \int _{\mathbb {R}^{(K + 1) \times C}} \\&\quad \exp \left( - \frac{1}{2} \zeta _{\text {w}} \Vert \textbf{W} \Vert _{F}^2 \right) d \textbf{W} \\&= - \frac{1}{2} \zeta _{\text {w}} \Vert \textbf{W} \Vert _{F}^2 - \log \left( \frac{2 \pi }{\zeta _{\text {w}}} \right) ^{0.5 \times (K + 1) \times C}. \end{aligned}$$

Therefore

$$\begin{aligned} \nabla _{\zeta _{\text {w}}} \log p \left( \textbf{W} \mid \zeta _{\text {w}} \right) = -0.5 \Vert \textbf{W} \Vert _{F}^2 + \frac{(K + 1) \times C}{2 \zeta _{\text {w}}} \end{aligned}$$

Having finally that

$$\begin{aligned} \nabla _{\zeta _{\text {w}}} p \left( \textbf{Y} \mid \tilde{\varvec{\Gamma }}^{\prime }, \zeta _{\text {w}} \right)&= \frac{(K + 1) \times C}{2 \zeta _{\text {w}}} - 0.5 \int _{\mathbb {R}^{(K + 1) \times C}}\\ &\quad \Vert \textbf{W} \Vert _{F}^2 p \left( \textbf{W} \mid \textbf{Y}, \tilde{\varvec{\Gamma }}^{\prime }, \zeta _{\text {w}} \right) d \textbf{W} \\&= \frac{(K + 1) \times C}{2 \zeta _{\text {w}}} - \frac{1}{2} \mathbb {E}_{\textbf{W} \mid \textbf{Y}, \tilde{\varvec{\Gamma }}^{\prime }, \zeta _{\text {w}}}\\ &\quad \left[ \Vert \textbf{W} \Vert _{F}^2 \right] , \end{aligned}$$

that is, the gradient we need for the iterative scheme in (8) depends on the computation of an expectation that can be estimated using MCMC methods. To approximate samples from the posterior distribution $p (\textbf{W} \mid \textbf{Y}, \tilde{\varvec{\Gamma }}^{\prime }, \zeta _{\text {w}} )$ and compute the latter expectation, the SOUL method uses the unadjusted Langevin algorithm (ULA) (Roberts and Tweedie 1996; Dalalyan 2017; Durmus and Moulines 2017), given by

9 $$\begin{aligned} W^{(k+1)} &  = W^{(k)} - \delta _{\text {ULA}} \nabla _W \log p (W^{(k)} \mid \textbf{Y}, \tilde{\varvec{\Gamma }}^{\prime }, \zeta _{\text {w}} )\nonumber \\  &  \quad + \sqrt{2 \delta _{\text {ULA}}} Z^{(n+1)} , \end{aligned}$$

where $\delta _{\text {ULA}} >0$ is a given step size and $(Z^{(n+1)})_{n \ge 0}$ is an i.i.d. sequence of $(K + 1) \times C$ - dimensional standard Gaussian random vectors. The SOUL method adapted for this problem, and details about its implementation can be found in Section C.

### Maximisation step regarding the variable $\textbf{W}$

Finally, the last expression to compute in the new SSLB model is given by

$$\begin{aligned} &  \widehat{\textbf{W}}=\underset{\textbf{W} \in \mathbb {R}^{(K+1) \times C}}{\operatorname {argmin}} \\ &  \quad \sum _{i=1}^N \sum _{l=1}^C y_{i l} \textbf{w}_l^T\left\langle \widetilde{\varvec{\gamma }}_i^{\prime }\right\rangle +\sum _{i=1}^N \Biggl \langle \log \left[ \sum _{l=1}^C \exp \left( \textbf{w}_l^T \widetilde{\varvec{\gamma }}_i^{\prime }\right) \right] \Biggr \rangle \\  &  \quad + \frac{1}{2} \hat{\zeta }_{\text {w}} \Vert \textbf{W} \Vert _{F}^2. \end{aligned}$$

Since there is no closed-form solution for the latter, we decided to implement an accelerated gradient descent (AGD) algorithm (Nesterov 1983; Güler 1992; Salzo and Villa 2012) to ensure rapid convergence to the minimum. To apply AGD, we need the gradient of the latter expression, which was given in Section D.1. This allows us to define the following iterative scheme

$$\begin{aligned} \textbf{W}^{(0)} &  = \textbf{W}^{(-1)} = \textbf{0} \in \mathbb {R}^{(K+1)\times C}; \; \textbf{V}^{(0)} \in \mathbb {R}^{(K+1)\times C}; \\ &  \quad \; t_0 = 0 \in \mathbb {R}, \end{aligned}$$

$$\begin{aligned} t_{s+1}&=\frac{1+\sqrt{1+4 t_s^2}}{2}, \\ \textbf{V}^{(s)}&=\textbf{W}^{(s)}+\frac{t_s-1}{t_{s+1}}\left( \textbf{W}^{(s)}-\textbf{W}^{(s-1)}\right) , \\ \textbf{W}^{(s+1)}&=\textbf{V}^{(s)} + \delta _{\text {AGD}} \overline{\nabla }_{\textbf{w}} \log p (\textbf{Y} \mid \tilde{\varvec{\Gamma }}^{\prime }, \textbf{V}^{(s)}, \hat{\zeta }_{\text {w}} ), \end{aligned}$$

$$\begin{aligned} s \in \{0,\ldots , S - 1\} \subset \mathbb {N}, \end{aligned}$$

where $\delta _{\text {AGD}}$ is the step size or learning rate of the iterative AGD scheme, which must be carefully set to avoid divergence. Note that, on each AGD iteration, we need to generate a collection of $\tilde{\varvec{\Gamma }}^{\prime (1)}, \ldots , \tilde{\varvec{\Gamma }}^{\prime (J)}$ samples from $p ( \widetilde{\gamma }_{ik}=1 \mid \textbf{Y}, \textbf{T}, \widetilde{\varvec{\theta }}, \textbf{W} )$ to compute the Monte Carlo estimate of the gradient (see Section D for details).

*Note:* While Section 3.4 approximates the posterior distribution of $\textbf{W}$ using the SOUL algorithm to estimate the $\ell _2$ regularisation hyperparameter, the current section focuses on computing a point estimate of $\textbf{W}$ via MAP optimisation within the M-step of the EM algorithm. These two steps serve distinct purposes within our inference framework: SOUL is used for hyperparameter tuning by integrating over $\textbf{W}$, while Nesterov’s method is employed for parameter estimation given fixed hyperparameters.

With the methodology established, we will refer to this modified version of the SSLB model as **OG-SSLB**, which stands for **Outcome-Guided Spike-and-Slab Lasso Biclustering**.

## Numerical Results

### Simulation Study

In this section, we evaluate the performance of OG-SSLB compared to SSLB in a simulation setting. We use the consensus score metric (Hochreiter et al. 2010) to measure the accuracy of biclusters identified by each method relative to the true biclusters. The highest possible consensus score is 1, indicating identical sets of biclusters.

We reproduce the simulation described in (Moran and George 2021, Section 3.1), where a simulated dataset with $N = 300$, $G = 1000$, and $K = 15$ biclusters is examined. The data simulation follows settings closely aligned with those in the FABIA (Hochreiter et al. 2010) and SSLB studies. The data matrix $\textbf{X}$ is generated as $\mathbf {Z \Lambda }^T + \textbf{E}$, with each entry in the noise matrix $\textbf{E}$ sampled from an independent standard normal distribution. For each column $\textbf{z}^k$, the number of samples in bicluster *k* is drawn uniformly from $\{5, \ldots , 20\}$. The indices of these elements are randomly selected and assigned values from $N(\pm 2, 1)$, with the sign of the mean chosen randomly. The elements of $\textbf{z}^k$ not in the biclusters have values drawn from $N(0, 0.2^2)$. The columns $\varvec{\lambda }^k$ are generated similarly, except that the number of elements in each bicluster is drawn from $\{10, \ldots , 50\}$.

To construct the disease outcome matrix $\textbf{Y}$ for the OG-SSLB algorithm, we first generate a matrix of weights $\textbf{W}$ in the following way

1. **Intercepts (Baseline Weights for Healthy Control Group)**: The first row of $\textbf{W}$, representing the intercepts, is initialised with small values

$$\begin{aligned} W_{1j} = \log (\epsilon ) \quad \text {for all } j, \end{aligned}$$

where $0< \epsilon < 1$. These small values correspond to the reference class (i.e., the healthy control group, HC) in the multinomial logistic regression model. In logistic regression, the intercept term controls the baseline probability of a sample belonging to the reference class when no covariates (in this case, the bicluster assignments) are active. By assigning a small value to $W_{1j}$, we increase the baseline probability for healthy control samples when no bicluster is assigned to a sample. Since $\log (\epsilon )$ with $\epsilon < 1$ results in a negative value, this translates into a higher probability of belonging to the reference class (HC).

2. **Weight Assignment for Biclusters (Bias Towards Disease Samples)**: For samples belonging to a bicluster, we adjust the weights in the remaining rows of $\textbf{W}$, corresponding to the non-reference classes (i.e., the disease classes). Specifically, we set the weights for the non-reference class as:

$$\begin{aligned} W_{ij} = \log (1 / \epsilon ) \quad \text {for } i>1, j . \end{aligned}$$

Here, $\log (1/\epsilon )$, where $\epsilon < 1$, results in a positive value, increasing the likelihood that samples assigned to a bicluster are classified as disease samples. Importantly, only one column $j$ is randomly selected for each row $i$ in $\textbf{W}$ to be assigned this value. This ensures that a specific bicluster is more strongly associated with a particular disease group, effectively biasing the model toward classifying samples in that bicluster as disease samples.

Finally, the assignment of disease for each sample is determined by applying the multinomial logistic regression model using the defined weights $\textbf{W}$.

The rationale behind making samples belonging to a bicluster more likely to be classified as disease cases stems from the biological assumption that disease states are often driven by specific gene expression patterns (Ota et al. 2021; Mesko et al. 2011; Veer et al. 2002; Vijver et al. 2002). Biclustering aims to identify subsets of genes that co-vary together in certain subsets of samples, which may represent distinct biological processes or pathways that are activated in disease conditions.

We adopt similar hyperparameter configurations for SSLB and OG-SSLB, detailed in (Moran and George 2021, Section 2.5). In particular, the slab parameters for the loadings and the factors, $\varvec{\Lambda }$ and $\textbf{Z}$, are set to $\omega _1, \tilde{\omega }_1 = 1$. The spike parameters for $\textbf{Z}$ follow an increasing sequence of $\omega _0 \in \{ 1, 5, 10, 50, 100, 500, 10^3, 10^4, 10^5, 10^6, 10^7 \}$. The spike parameters for $\textbf{Z}$ are chosen as $\tilde{\omega }_0 \in \{ 1, 5, \ldots , 5 \}$ to correspond to the length of the sequence $\omega _0$. Specifically, the values of $\tilde{\omega }_0$ are fixed at $\tilde{\omega }_0 = 5$. Furthermore, the initial overestimate of the number of biclusters is set to $K^* = 30$.

We compared 50 realisations of SSLB and OG-SSLB using the same simulated dataset across all runs while varying the algorithmic initial conditions for each of the fifty runs. This analysis was performed under three distinct implementations: SSLB/OG-SSLB with the Pitman–Yor extension (PY), where $\tilde{\alpha } = 1$ and $d = 0.5$, SSLB/OG-SSLB with the stick-breaking IBP prior (IBP) where $\tilde{\alpha } = 1$, and SSLB/OG-SSLB with the finite approximation to the IBP prior (Beta-Binomial, BB), where $\tilde{a} = 1/K^*$ and $\tilde{b} = 1$. For OG-SSLB, we implemented two variations: a non-informative approach, which assigns values around $\log (1)$ to all elements of the matrix $\textbf{W}$, resulting in an imprecise simulated $\textbf{Y}$ outcome, and an informative approach, which assigns $\log (1/2)$ to the intercepts and $\log (4)$ to specific bicluster-disease elements (i.e., one specific column in each row of $\textbf{W}$), yielding a more informative simulated $\textbf{Y}$ outcome. We aim to show the difference in adding more information to the model.

**Fig. 4:**
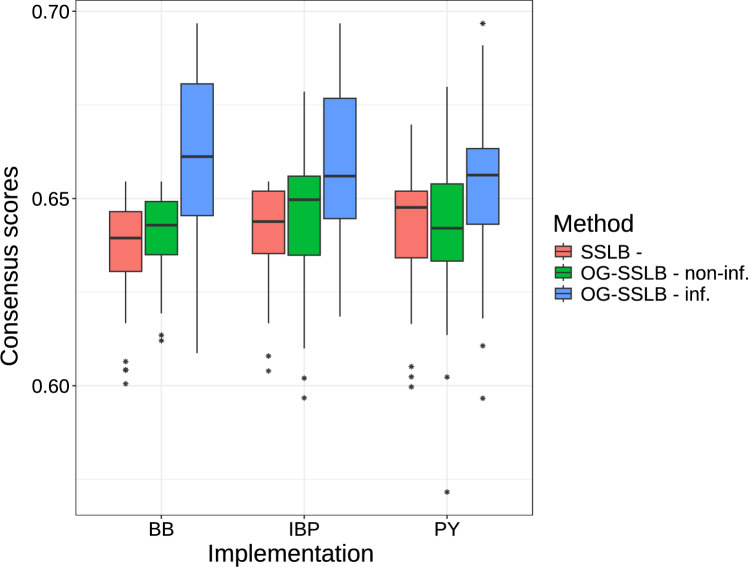
Consensus scores of 50 replications of SSLB and OG-SSLB, both using three different prior implementations for $\tilde{\varvec{\Gamma }}$: BB, PY and IBP (see Section 2.1.2 for details). For OG-SSLB, we implement a non-informative (i.e., non-inf.) approach, setting values for all elements of the matrix $\textbf{W}$ around $\log (1)$, which produces a diffusive $\textbf{Y}$ outcome, and an informative (i.e., inf.) approach, where we set $\log (1/2)$ for the intercepts and $\log (4)$ for specific bicluster-disease elements in the matrix $\textbf{W}$, resulting in a more informative $\textbf{Y}$ outcome.

**Fig. 5:**
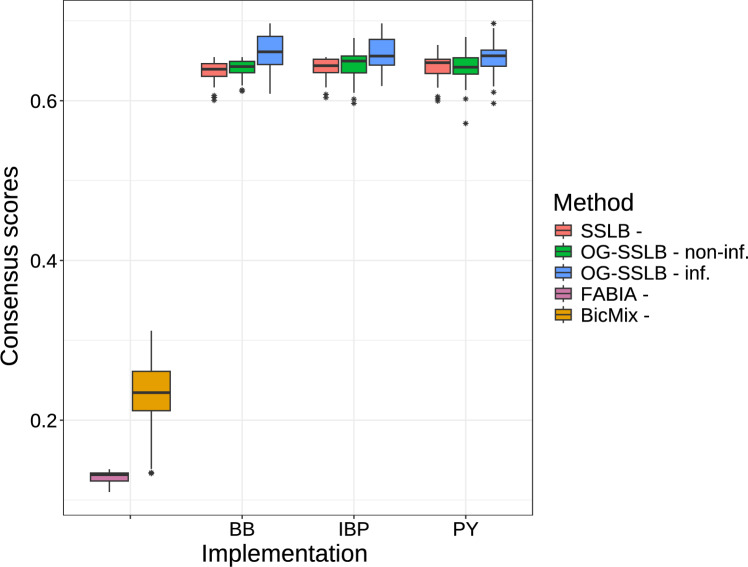
Consensus scores of 50 replicates for FABIA, BicMix, SSLB, and OG-SSLB. For (OG-)SSLB, we include results under the three prior choices for $\tilde{\varvec{\Gamma }}$: BB, PY, and IBP (see Section 2.1.2). OG-SSLB results are shown for both the non-informative and informative settings described in the caption of Figure 4.

The distribution of the consensus score for each method can be seen in Figure 4. OG-SSLB consistently achieves higher consensus scores in all three prior versions of the binary indicators for the factors $\textbf{Z}$, reaching even higher precision in the informative case (with a slight increase in the PY prior version for the informative case). We also illustrated in Figure 5 a comparison of the SSLB and OG-SSLB methods with the FABIA and BicMix algorithms. It is important to mention that FABIA requires the number of biclusters in advance, so we provided the true number of biclusters (i.e., $k=15$) in all 50 runs. BicMix has its own method for estimating the number of biclusters in the data (Gao et al. 2016). As shown in the figure, the consensus scores achieved by FABIA and BicMix are considerably lower than those of SSLB and OG-SSLB. In addition, Table 1 presents the mean, over the 50 runs, of the estimated number of biclusters $\hat{K}$ for the methods that estimate *K*. All implementations of the informative OG-SSLB approach are closer to the true number of biclusters compared to SSLB. BicMix achieves a mean estimate of 12.56 biclusters. To further validate our results, we also evaluated biclustering accuracy using the Clustering Error metric (Horta and Campello 2014; Nicholls and Wallace 2021). These results, presented in Appendix E, confirm the same trend observed with the consensus score, with OG-SSLB (inf.) consistently achieving the best performance.

Regarding the corresponding run times, the SSLB implementations required between 35 and 60 seconds, whereas the OG-SSLB implementations took between 2600 and 3500 seconds. While OG-SSLB is computationally more intensive due to the additional stochastic optimisation steps for estimating regularisation parameters due to the SOUL algorithm, this increase is justified by the significant gains in performance, particularly in identifying more stable and biologically meaningful biclusters. These results demonstrate a clear trade-off between computational cost and modelling flexibility. We discuss potential strategies to mitigate this cost in the Conclusion.

**Table 1:** Mean estimated number of biclusters, $\hat{K}$, over 50 replications ($K_{\text {true}}=15$). The three SSLB implementations (BB, IBP, PY) refer only to SSLB and OG-SSLB.

**SSLB**	**Method**			
**implementation**	**SSLB**	**OG-SSLB (non-inf.)**	**OG-SSLB (inf.)**	**BicMix**
BB	14.04	14.02	14.50	$\vert$
IBP	14.10	14.36	14.50	12.56
PY	14.34	14.42	14.46	$\vert$

### Breast Cancer Microarray Dataset

The dataset used in this study consists of gene expression data from 337 breast cancer patients diagnosed with stage I or II breast cancer (Vijver et al. 2002; Veer et al. 2002). It comprises the expression levels of 24,158 genes, resulting in a large and high-dimensional data structure. These data have been widely used to study the heterogeneity of breast cancer, a disease known to comprise several molecular subtypes, including estrogen receptor-positive (ER+) and estrogen receptor-negative (ER-) subtypes. The patients’ ER status is determined based on the expression of the ESR1 gene, which encodes the estrogen receptor, and serves as a key indicator of the disease subtype and treatment options.

The goal of this analysis is to identify meaningful biclusters of patients and genes that reflect the biological differences between these subtypes, particularly focusing on the estrogen receptor status (ER+ or ER-). Biclustering is especially well-suited for this task as it allows the simultaneous grouping of patients and relevant genes, which may highlight subtype-specific gene expression patterns. Consequently, it has also been used as a benchmark for biclustering methods such as FABIA (Hochreiter et al. 2010), BicMix (Gao et al. 2016), and SSLB.

To assess the performance of our proposed OG-SSLB method compared to the standard SSLB algorithm, we ran 50 replicates, each with different initial conditions, for both methods. Both SSLB and OG-SSLB were initialised with an overestimate of the number of biclusters $K^* = 50$. For the loadings, $\varvec{\Lambda }$, we configure the Beta-Binomial hyperparameters as $a=1/(GK^*)$ and $b=1$. This normalisation by *G* enhances the focus on sparsity. For the factors, $\textbf{Z}$, the IBP prior is used with hyperparameters set to $\tilde{\alpha } = 1/N$ and $d=0$. The other parameters are assigned the default values specified in (Moran and George 2021, Section 2.5). For the OG-SSLB method, we incorporate the patients’ ER status (1 for ER+ and 0 for ER-) as the outcome variable.

**Fig. 6:**
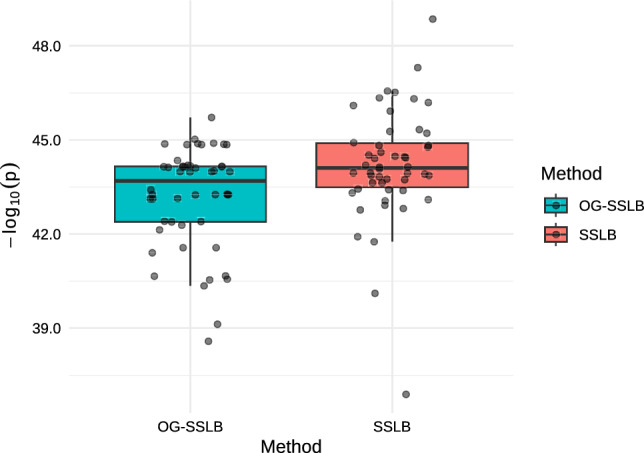
Distribution of the smallest p-value from Wilcoxon rank-sum tests comparing ER+ vs ER– patients across estimated factors (the estimated $\textbf{Z}$ matrix) for each estimated bicluster in each of the 50 SSLB and OG-SSLB replicates applied to the breast cancer microarray data.

After obtaining the biclusters, we performed a Wilcoxon rank-sum test on the estimated factors (i.e., $\textbf{Z}$ matrix) for each bicluster in every replicate, comparing the distributions of factor values between ER+ and ER– patients. For each run, we recorded the minimum p-value across all biclusters. The resulting $-\log _{10}(p)$ values are summarised in Figure 6. As shown, both SSLB and OG-SSLB achieve similarly high significance across replicates, suggesting that in this real-data scenario, both methods are equally able to recover subtype-relevant biclusters.

### Immune Cell Gene Expression Atlas, University of Tokyo

Detecting biclusters in transcriptomic data is one of the motivating applications for OG-SSLB, therefore, we also applied the SSLB and OG-SSLB methods to gene expression data from Ota et al. (2021). This study provides a comprehensive database of transcriptomic and genome sequencing data from a wide range of immune cells from patients with immune-mediated diseases (IMD). This collection of data, termed the “Immune Cell Gene Expression Atlas from the University of Tokyo (ImmuNexUT)", includes gene expression patterns consisting of healthy volunteers and patients diagnosed with systemic lupus erythematosus (SLE), idiopathic inflammatory myopathy (IIM), systemic sclerosis (SSc), mixed connective tissue disease (MCTD), Sjögren’s syndrome (SjS), rheumatoid arthritis (RA), Behçet’s disease (BD), adult-onset Still’s disease (AOSD), ANCA-associated vasculitis (AAV), or Takayasu arteritis (TAK). The dataset encompasses 28 distinct immune cell types, nearly covering all peripheral immune cells. We anticipate that a subset of genes may cluster in a subset of patients who share some specific aetiology, and that this bicluster will be enriched in genes related to that aetiology and patients with related diseases.

In order to evaluate our method in a real-world dataset where we have some expectation of what to find, we focused on monocytes, which are known to express an interferon-response gene expression signature, found more often in patients with IMD, and particularly SLE (Nikolakis et al. 2023; Perez et al. 2022). The data were pre-processed as follows:

1. We perform batch normalisation using the ComBat-seq R package (Zhang et al. 2020).
2. We reduce low-count genes using the edgeR package (Robinson et al. 2009).
3. We calculated the Pearson correlation matrix between genes and setting a threshold at the 90th percentile, we focus on the most highly correlated gene pairs. Genes with fewer than five other genes correlating above this threshold are removed to eliminate those with weak or non-specific interactions, which could be noisy or less informative. This step ensures that only genes with strong co-expression relationships, potentially reflecting meaningful biological connections, are retained.
4. Finally, to correct for technical variation and differences in sequencing depth between samples, we applied the median of ratios normalisation method, as implemented in the DESeq2 R package (Love et al. 2014). This normalisation ensures that gene expression differences reflect true biological variability rather than artefacts from varying read counts across samples.

Following preprocessing, we obtained a dataset comprising $N = 410$ and $G = 11215$. We ran 20 different replicates of both SSLB and OG-SSLB using the IBP prior for $\tilde{\varvec{\Gamma }}$, with hyperparameters $\tilde{\alpha } = 1/N$ and $d=0$, and the Beta-Bernoulli prior hyperparameters for $\varvec{\Gamma }$ set to $a = 1 / (G K^*)$ and $b = 1$. Our choice of using the IBP prior and the specified hyperparameters values for the factor and loading binary indicator matrices is in agreement with the real data experiments performed in the SSLB paper (see (Moran and George 2021, Sections 4 and 5) for details). The remaining hyperparameter settings were similarly aligned with those of the previous numerical experiment. Furthermore, the initial overestimate for the number of biclusters was set to $K^* = 50$.

From Nicholls et al. (2022), we obtained a list of 56 genes associated with the IFN signature, 51 of which were found present in the preprocessed dataset. To focus on sparse, IFN-related biclusters, we filtered the results from both methods to include only biclusters with less than 50% of the total number of samples and more than 6 IFN genes.

**Fig. 7:**
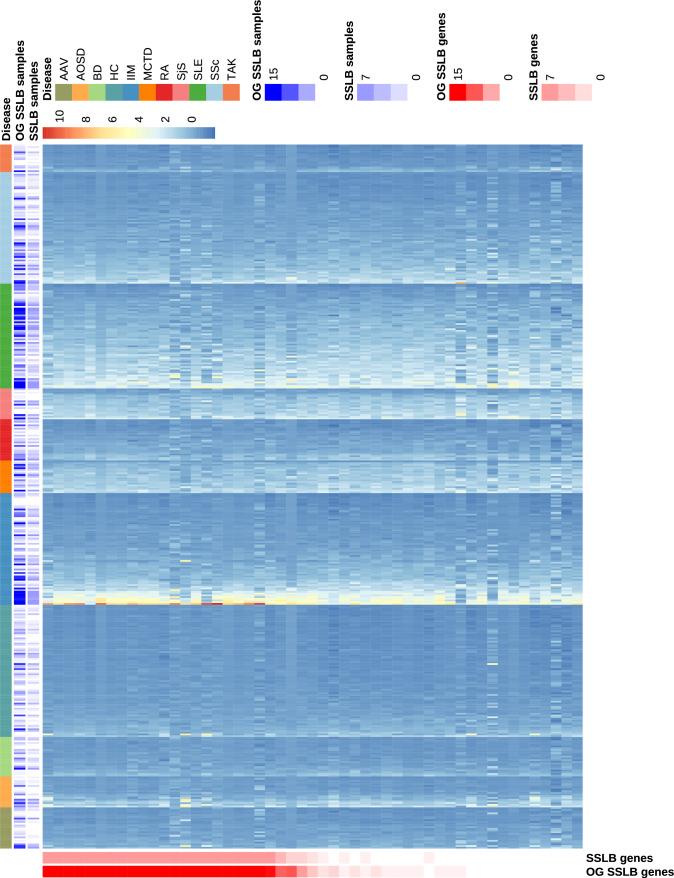
Heatmap of standardised gene expression data from the ImmuNexUT study, where each column represents a sample and each row corresponds to one of the 51 interferon (IFN) signature genes. The data has been centred and scaled. The colour bars on the margins indicate, across 20 runs, how frequently each sample or gene was included in any sparse IFN-related bicluster identified by OG-SSLB or SSLB. This is not a single bicluster but an aggregate visualisation to summarise common inclusion patterns. Samples are grouped by disease.

The results are first summarised in Figure 7, where a heatmap of the standardised gene expression data is shown. As can be seen, SSLB and OG-SSLB generally identify the same samples and genes forming the IFN biclusters, but OG-SSLB does so far more consistently: it finds IFN-related biclusters ($\ge 7$ of the 51 known IFN genes) in 18 of 20 runs, whereas SSLB does so in only 7 of 20 runs.

Note that Figure 7 does not show a single bicluster, but instead summarises the frequency with which each sample and IFN gene is selected into any of the sparse IFN-related biclusters detected across 20 runs of each method. The bar annotations along the margins of the heatmap reflect how often each gene or sample appears in at least one such bicluster for OG-SSLB or SSLB, respectively. This aggregated view helps visualise consistent patterns across replicates rather than illustrating a single bicluster instance.

These results have also been summarised in Table 2. As can be seen, OG-SSLB produces a substantially larger number of replicates in which sparse IFN-related biclusters were detected, in comparison to SSLB.

**Table 2:** Results from 20 replicates of applying SSLB and OG-SSLB to the ImmuNexUT real data, focusing on sparse IFN-related biclusters (< 50% of samples, > 6 IFN genes).

Method	Replicates with at least one bicluster that meets sparse filtering cond.	Median % SLE patients in bicluster’s replicates
SSLB	7	36
OG-SSLB	18	43.5

**Fig. 8:**
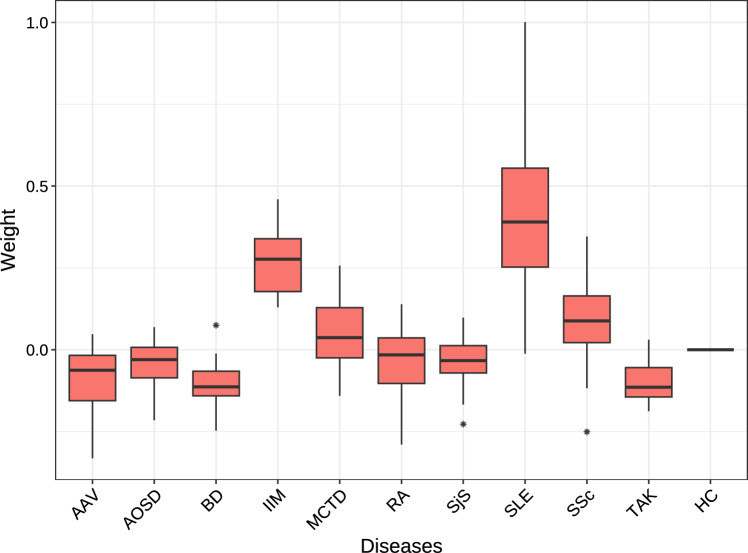
Distribution of the estimated weight $\textbf{W}$ values by OG-SSLB, for each disease, in sparse IFN-related biclusters (i.e., biclusters with less than 50% of the total samples and more than 6 IFN genes).

To further examine disease associations, we analysed the matrix of weights $\textbf{W}$ estimated by OG-SSLB, focused only on the biclusters corresponding to the sparse IFN-related condition. As shown in Figure 8, SLE consistently exhibited the strongest weights across these biclusters, indicating that these IFN-related profiles are more pronounced in SLE patients compared to other disease groups. Notably, idiopathic inflammatory myopathies (IIM) also showed strong weight values for the sparse IFN-related clusters. This agrees with the known role of type 1 interferon in IIM (Lundberg and Helmers 2010) and its association with disease activity (Kamperman et al. 2024).

**Fig. 9:**
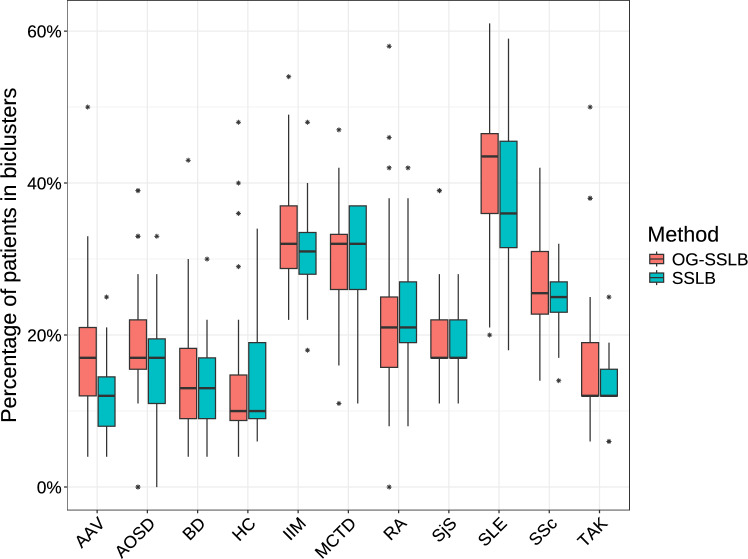
Results from 20 replicates of applying SSLB and OG-SSLB to the ImmuNexUT real data, focusing on biclusters with less than 50% of the total samples and more than 6 IFN genes: Distribution of the percentage of samples per disease.

**Fig. 10:**
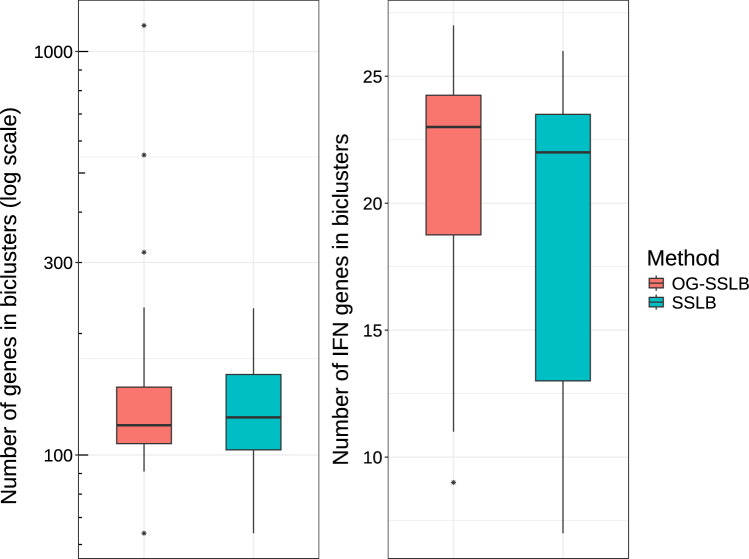
Results from 20 replicates of applying SSLB and OG-SSLB to the ImmuNexUT real data, focusing on biclusters with less than 50% of the total samples and more than 6 IFN genes. Left: Distribution of the total number of genes in biclusters identified by both methods. Right: Distribution of the number of IFN gene signatures in biclusters identified by both methods.

Furthermore, Figures 9 and 10 show the distribution of samples and genes identified by the SSLB and OG-SSLB runs. Although neither method recovers all 51 IFN genes in a single bicluster, OG-SSLB identified biclusters under the specified conditions exhibit, in distribution, a higher percentage of SLE patients and a higher number of IFN gene signatures. Additionally, while SLE patients exhibited the highest fraction of patients in the IFN biclusters, IFN signatures have been found in other IMD, and both methods found a higher fraction of patients in IFN biclusters for IIM, MCTD, RA, SjS and SSc. Concerning the associated run times, the SSLB algorithm required about 1900 seconds to run, whereas the OG-SSLB algorithm took approximately 44700 seconds.

## Conclusions and Discussion

In conclusion, our proposed algorithm, OG-SSLB, exhibits superior performance compared to the SSLB approach in both numerical and real-data experiments, particularly in its ability to estimate the number of biclusters more accurately and achieve higher consensus scores. The flexibility of OG-SSLB, particularly through its multinomial modelling framework, allows it to accommodate more complex clustering structures than the commonly used binomial models. While this improvement entails significantly higher computational costs due to iterative processes such as AGD and ULA, the enhanced precision and modelling capacity of OG-SSLB make it a valuable contribution to biclustering methodologies.

Our subsequent analyses will expand the OG-SSLB framework to the ImmuNexUT dataset, investigating other cell types and detecting new gene expression signatures rather than focusing solely on predefined ones. We aim to identify similarities between diseases, facilitated by the bicluster overlapping allowed by this method.

Additionally, we will explore other machine learning alternatives to multinomial logistic regression, such as support vector machines and naive Bayes, which may offer more robust solutions for integrating disease information into the biclustering framework. An especially promising approach could be the introduction of deep learning classifiers, which may better capture the potential non-linearity of boundary classes, thereby further enhancing the quality of the incorporated disease information. While deep learning classifiers can identify more complex patterns in the data, they would require significantly larger computational resources compared to the multinomial logistic regression model. Nonetheless, the shift from multinomial logistic regression to deep learning or other machine learning approaches presents exciting opportunities to improve classification accuracy while addressing the challenges inherent in genomic data analysis.

We acknowledge that OG-SSLB incurs greater computational overhead compared to SSLB, due primarily to the iterative SOUL-based estimation of regularisation parameters in the multinomial regression framework. However, this added complexity enables more precise integration of outcome information and results in more informative biclusters. Future work will also explore strategies to reduce this execution time. For example, a practical approach might involve limiting the number of EM iterations during which the SOUL algorithm is applied and subsequently fixing the regularisation hyperparameters using the average of their recent estimates. This would avoid the need to rerun SOUL in later iterations. Although this is beyond the scope of the present article, this line of investigation may offer a promising trade-off between computational efficiency and model performance.

Regarding future applications, OG-SSLB could play a meaningful role in personalised medicine and biomarker discovery. By identifying sparse and interpretable biclusters that capture subgroups of patients and genes associated with specific disease outcomes, OG-SSLB may help uncover clinically relevant gene signatures. These could serve as potential diagnostic markers or guide stratification of patients for targeted therapies. In particular, its ability to incorporate disease labels makes OG-SSLB especially well-suited to reveal molecular patterns linked to disease heterogeneity, offering translational insights in clinical research.

The source code to reproduce the results in this paper is available online at https://github.com/luisvargasmieles/OGSSLB-examples.

## Figures and Tables

**Algorithm 1:**
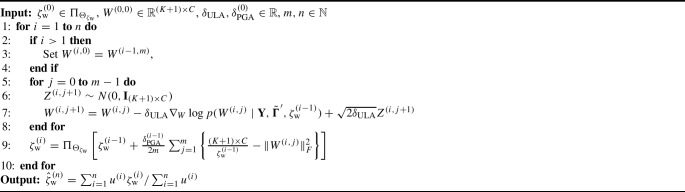
SOUL algorithm for the estimation of $\zeta _{\text {w}}$

**Fig. 11:**
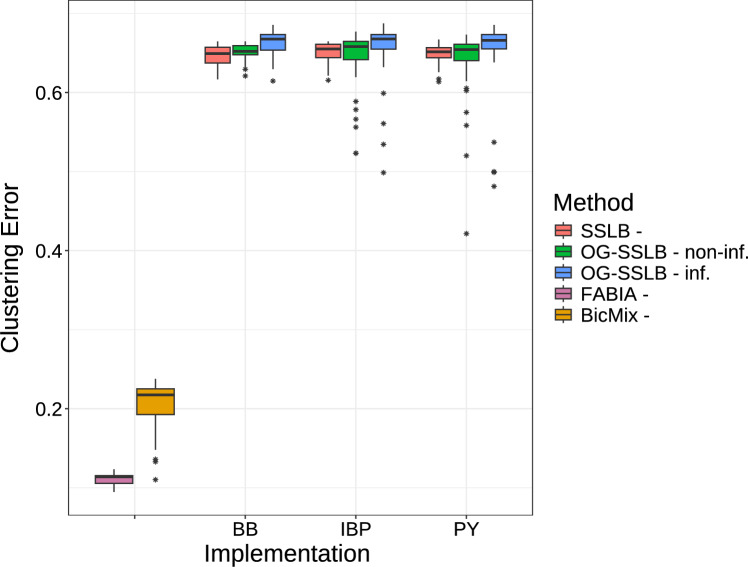
Clustering error of 50 replicates for FABIA, BicMix, SSLB, and OG-SSLB. For (OG-)SSLB, we include results under the three prior choices for $\tilde{\varvec{\Gamma }}$: BB, PY, and IBP (see Section 2.1.2). OG-SSLB results are shown for both the non-informative and informative settings described in Section 4.1 and in the caption of Figures 4.

## Data Availability

The implementation of the OGSSLB algorithm can be found as an R/C++ package at https://github.com/luisvargasmieles/OGSSLB. The code that supports the findings of this paper is available on the GitHub page: https://github.com/luisvargasmieles/OGSSLB-examples. The dataset used for the Breast Cancer Microarray experiment is publicly available in the R package breastCancerNKI. The dataset for the Immune Cell Gene Expression Atlas (ImmuNexUT) experiment is also in the public domain and is available at the National Bioscience Database Center (NBDC), with the study accession code E-GEAD-397.

## Appendix A Current SSLB model: E step

For completeness, we present the log posterior and the related expression for the E-step of SSLB. For additional details, refer to Moran and George (2021). The complete log posterior is as follows

A1 $$\begin{aligned}&\log p(\varvec{\Delta }, \textbf{Z}, \tilde{\mathbf {\Gamma }} \mid \textbf{X} ) \propto -\frac{1}{2} \sum _{i=1}^N\left\{ \left( \textbf{x}_{\textbf{i}}-\textbf{z}_{\varvec{i}}\varvec{\Lambda }^T\right) \right. \nonumber \\&\quad \left. \mathbf {\Sigma }^{-1}\left( \textbf{x}_{\textbf{i}}-\textbf{z}_{\varvec{i}}\varvec{\Lambda }^T\right) ^T\right\} \nonumber \\&\quad - \frac{N+\eta +2}{2} \sum _{j=1}^G \log \sigma _j^2 -\sum _{j=1}^G \frac{\eta \xi }{2 \sigma _j^2} -\sum _{k=1}^{K^*} \log p \left( \varvec{\lambda }^{\textbf{k}}\right) \nonumber \\ &\quad -\frac{1}{2} \sum _{i=1}^N \textbf{z}_{\textbf{i}} \textbf{D}_{\textbf{i}} \textbf{z}_{\textbf{i}}^T -\frac{1}{2} \sum _{i=1}^N \sum _{k=1}^{K^*} \log \tau _{i k} \nonumber \\&\quad -\frac{1}{2} \log \sum _{i=1}^N \sum _{k=1}^{K^*}\left[ \left( 1-\tilde{\gamma }_{i k}\right) \widetilde{\omega }_0 \exp \left( - \frac{\widetilde{\omega }_0 \tau _{i k}}{2}\right) \right. \nonumber \\&\quad \left. +\tilde{\gamma }_{i k} \widetilde{\omega }_1 \exp \left( - \frac{\widetilde{\omega }_1 \tau _{i k}}{2} \right) \right] \nonumber \\&\quad +\sum _{k=1}^{K^*}\left[ \left( \sum _{i=1}^N \tilde{\gamma }_{i k}\right) \log \prod _{l=1}^k \nu _l+\left( N-\sum _{i=1}^N \tilde{\gamma }_{i k}\right) \right. \nonumber \\&\quad \left. \log \left( 1-\prod _{l=1}^k \nu _l\right) \right] +(\tilde{\alpha }-1) \sum _{k=1}^{K^*} \log \nu _k, \end{aligned}$$

where $\textbf{D}_{\textbf{i}}=\operatorname {diag}\left\{ \tau _{i 1}^{-1}, \ldots , \tau _{i K^*}^{-1}\right\}$ and $\varvec{\Gamma }$, $\varvec{\theta }$ have been margined out to have $p (\varvec{\Lambda })$ (see Ročková and George (2018) for details). This allows us to define

A2 $$\begin{aligned}&Q(\varvec{\Delta } \mid \varvec{\Delta }^{(t)}) \propto -\frac{1}{2} \sum _{i=1}^N \left\{ \left( \textbf{x}_{i} - \left\langle \textbf{z}_{i} \right\rangle \varvec{\Lambda }^T \right) \varvec{\Sigma }^{-1} \left( \textbf{x}_{i} - \left\langle \textbf{z}_{i} \right\rangle \varvec{\Lambda }^T \right) ^T \right. \nonumber \\&\left. + \operatorname {tr} \left[ \varvec{\Lambda }^T \varvec{\Sigma }^{-1} \varvec{\Lambda } \left( \left\langle \textbf{z}_{i} \textbf{z}_{i}^T \right\rangle - \left\langle \textbf{z}_{i} \right\rangle \left\langle \textbf{z}_{i} \right\rangle ^T \right) \right] \right\} \nonumber \\&-\frac{N+\eta +2}{2} \sum _{j=1}^G \log \sigma _j^2 - \sum _{j=1}^G \frac{\eta \xi }{2 \sigma _j^2} - \sum _{k=1}^{K^*} \log p \left( \varvec{\lambda }^{k} \right) \nonumber \\&-\frac{1}{2} \sum _{i=1}^N \left\{ \left\langle \textbf{z}_{i} \right\rangle \textbf{D}_{i} \left\langle \textbf{z}_{i} \right\rangle ^T + \operatorname {tr} \left[ \textbf{D}_{i} \left( \left\langle \textbf{z}_{i} \textbf{z}_{i}^T \right\rangle - \left\langle \textbf{z}_{i} \right\rangle \left\langle \textbf{z}_{i} \right\rangle ^T \right) \right] \right\} \nonumber \\&-\frac{1}{2} \sum _{i=1}^N \sum _{k=1}^{K^*} \log \tau _{i k} -\frac{1}{2} \sum _{i=1}^N \sum _{k=1}^{K^*} \left[ \left( 1 - \left\langle \tilde{\gamma }_{i k} \right\rangle \right) \widetilde{\omega }_0^2 + \left\langle \tilde{\gamma }_{i k} \right\rangle \widetilde{\omega }_1^2 \right] \tau _{i k} \nonumber \\&+\sum _{k=1}^{K^*} \left[ \left( \sum _{i=1}^N \left\langle \tilde{\gamma }_{i k} \right\rangle \right) \nonumber \right. \\&\left. \log \prod _{l=1}^k v_l + \left( N - \sum _{i=1}^N \left\langle \tilde{\gamma }_{i k} \right\rangle \right) \log \left( 1 - \prod _{l=1}^k v_l \right) \right] \nonumber \\&+(\tilde{\alpha } - 1) \sum _{k=1}^{K^*} \log v_k , \end{aligned}$$

where $$\mathbb {E}_{\textbf{Z}, \widetilde{\Gamma } \mid \Delta ^{(t)}, \textbf{X}}[W] = \langle W\rangle $$.

## Appendix B SSLB model with profile regression: E step

The log posterior for the SSLB model using profile regression can be expressed as follows

B3 $$\begin{aligned}&\log p(\varvec{\Delta }, \textbf{Z}, \tilde{\mathbf {\Gamma }} \mid \textbf{X}, \textbf{Y} ) \propto -\frac{1}{2}\nonumber \\&\quad \sum _{i=1}^N\left\{ \left( \textbf{x}_{\textbf{i}}-\textbf{z}_{\varvec{i}}\varvec{\Lambda }^T\right) \mathbf {\Sigma }^{-1}\left( \textbf{x}_{\textbf{i}}-\textbf{z}_{\varvec{i}}\varvec{\Lambda }^T\right) ^T\right\} \nonumber \\&\quad -\frac{N+\eta +2}{2} \sum _{j=1}^G \log \sigma _j^2-\sum _{j=1}^G \frac{\eta \xi }{2 \sigma _j^2} \nonumber \\&\quad -\sum _{k=1}^{K^*} \log p \left( \varvec{\lambda }^{\textbf{k}}\right) -\frac{1}{2} \sum _{i=1}^N \textbf{z}_{\textbf{i}} \textbf{D}_{\textbf{i}} \textbf{z}_{\textbf{i}}^T-\frac{1}{2} \sum _{i=1}^N \sum _{k=1}^{K^*} \log \tau _{i k} \nonumber \\&\quad -\frac{1}{2} \log \sum _{i=1}^N \sum _{k=1}^{K^*}\left[ \left( 1-\tilde{\gamma }_{i k}\right) \widetilde{\omega }_0 \exp \left( - \frac{\widetilde{\omega }_0 \tau _{i k}}{2}\right) \right. \nonumber \\&\quad \left. +\tilde{\gamma }_{i k} \widetilde{\omega }_1 \exp \left( - \frac{\widetilde{\omega }_1 \tau _{i k}}{2} \right) \right] \nonumber \\&\quad +\sum _{k=1}^{K^*}\left[ \left( \sum _{i=1}^N \tilde{\gamma }_{i k}\right) \log \prod _{l=1}^k \nu _l \right. \nonumber \\&\left. + \left( N-\sum _{i=1}^N \tilde{\gamma }_{i k}\right) \log \left( 1-\prod _{l=1}^k \nu _l\right) \right] \nonumber \\&\quad + (\tilde{\alpha }-1) \sum _{k=1}^{K^*} \log \nu _k \nonumber \\&\quad -\sum _{i=1}^N \sum _{l=1}^C y_{i l}\textbf{w}^{(l)\,T} \tilde{\varvec{\gamma }}_i^{\prime }+\sum _{i=1}^N \log \left[ \sum _{l=1}^C \exp \left( \textbf{w}^{(l)\,T} \tilde{\varvec{\gamma }}_i^{\prime }\right) \right] \nonumber \\&\quad + \frac{1}{2} \zeta _{\text {w}} \Vert \textbf{W} \Vert _{F}^2. \end{aligned}$$

Hence, the corresponding equation for the E-step is now defined as

B4 $$\begin{aligned}&Q(\varvec{\Delta } \mid \varvec{\Delta }^{(t)}) \propto {-}\frac{1}{2} \sum _{i{=}1}^N\left\{ \left( \textbf{x}_{i} {-} \left\langle \textbf{z}_{i} \right\rangle \varvec{\Lambda }^T \right) \varvec{\Sigma }^{{-}1} \left( \textbf{x}_{i} {-} \left\langle \textbf{z}_{i} \right\rangle \varvec{\Lambda }^T \right) ^T \right. \nonumber \\&\quad \left. + \operatorname {tr}\left[ \varvec{\Lambda }^T \varvec{\Sigma }^{-1} \varvec{\Lambda } \left( \left\langle \textbf{z}_{i} \textbf{z}_{i}^T \right\rangle - \left\langle \textbf{z}_{i} \right\rangle \left\langle \textbf{z}_{i} \right\rangle ^T \right) \right] \right\} \nonumber \\&\quad -\frac{N+\eta +2}{2} \sum _{j=1}^G \log \sigma _j^2-\sum _{j=1}^G \frac{\eta \xi }{2 \sigma _j^2} -\sum _{k=1}^{K^*} \log p \left( \varvec{\lambda }^{\textbf{k}}\right) \nonumber \\&\quad -\frac{1}{2} \sum _{i=1}^N\left\{ \left\langle \textbf{z}_{i} \right\rangle \textbf{D}_{i} \left\langle \textbf{z}_{i} \right\rangle ^T + \operatorname {tr} \left[ \textbf{D}_{i} \left( \left\langle \textbf{z}_{i} \textbf{z}_{i}^T \right\rangle - \left\langle \textbf{z}_{i} \right\rangle \left\langle \textbf{z}_{i} \right\rangle ^T \right) \right] \right\} \nonumber \\&\quad - \frac{1}{2} \sum _{i=1}^N \sum _{k=1}^{K^*} \log \tau _{i k}\nonumber \\&\quad -\frac{1}{2} \sum _{i=1}^N \sum _{k=1}^{K^*}\left[ \left( 1-\left\langle \tilde{\gamma }_{i k}\right\rangle \right) \widetilde{\omega }_0^2+\left\langle \tilde{\gamma }_{i k}\right\rangle \widetilde{\omega }_1^2\right] \tau _{i k} \nonumber \\&\quad +\sum _{k=1}^{K^*}\left[ \left( \sum _{i=1}^N\left\langle \tilde{\gamma }_{i k}\right\rangle \right) \log \prod _{l=1}^k v_l \right. \nonumber \\&\quad + \left. \left( N-\sum _{i=1}^N\left\langle \tilde{\gamma }_{i k}\right\rangle \right) \log \left( 1-\prod _{l=1}^k v_l\right) \right] \nonumber \\&\quad +(\tilde{\alpha }-1) \sum _{k=1}^{K^*} \log v_k -\sum _{i=1}^N \sum _{l=1}^C y_{i l} \textbf{w}^{(l)\,T}\left\langle \widetilde{\varvec{\gamma }}_i^{\prime }\right\rangle \nonumber \\&\quad + \sum _{i=1}^N \Biggl \langle \log \left[ \sum _{l=1}^C \exp \left( \textbf{w}^{(l)\,T} \widetilde{\varvec{\gamma }}_i^{\prime }\right) \right] \Biggr \rangle + \frac{1}{2} \zeta _{\text {w}} \Vert \textbf{W} \Vert _{F}^2, \end{aligned}$$

and the variables at which the new $Q(\varvec{\Delta } \mid \varvec{\Delta }^{(t)})$ will be maximised in the M step are now $\varvec{\Delta }=\{\textbf{Z}, \varvec{\Sigma }, \textbf{T}, \textbf{W}, \nu \}$.

## Appendix C SOUL algorithm: further details

After defining in Section 3.4 the mathematical structure of the steps involved in estimating the hyperparameter $\zeta _{\text {w}}$ for the $\ell _2$ regularisation term in (5), below we present the SOUL algorithm.

### C.1 SOUL implementation guidelines

The implementation guidelines and details about the SOUL algorithm can be found in De Bortoli et al. (2021) and in (Vidal et al. 2020, Section 3.3). For completeness, we will provide some details below.

#### C.1.1 Setting $\delta _{\text {PGA}}^i$ and *m*

It is suggested in Vidal et al. (2020) to set $\delta _{\text {PGA}}^{(i)} = C_0 i^{-p}$ where *p* is within the range [0.6, 0.9] (in our experiments, we set $p=0.8$) and $C_0 \in \mathbb {R}$ a constant that can be initially set as $(\zeta _{\text {w}}^{(0)} \times (K + 1) \times C)^{-1}$ and adjusted as needed. For *m*, we followed the recommendation in De Bortoli et al. (2021); Vidal et al. (2020) using a single sample per iteration (that is, $m=1$), as we did not observe significant differences with larger values of *m*.

#### C.1.2 Setting $u^{(i)}$

According to the guidelines in Vidal et al. (2020), it is recommended to set $u^{(i)}$ as follows:

$$\begin{aligned} u^{(i)} = {\left\{ \begin{array}{ll} 0 & \text {if } i < N_0, \\ 1 & \text {if } N_0 \le i \le n, \\ \end{array}\right. } \end{aligned}$$

where $N_0 \in \mathbb {N}$ is the number of initial iterations to be discarded to reduce non-asymptotic bias, which corresponds to a burn-in stage. The range $i \in [N_0, n]$ represents the estimation phase of the averaging where the values of $\zeta _{\text {w}}^{(i)}$ have reached convergence and stabilised. In the interest of computational efficiency, we have set in our experiments $N_0 = 75$ and $n = 150$.

#### C.1.3 Implementation in Logarithmic Scale

The proposed methods for estimating $\zeta _{\text {w}}$ generally achieve better numerical convergence when implemented on a logarithmic scale, as recommended in (Vidal et al. 2020, Section 3.3.2). Therefore, we apply a variable transformation $\kappa = \log (\zeta _{\text {w}})$, estimate $\hat{\kappa }$ using the SOUL algorithm, and then determine $\hat{\zeta }_{\text {w}} = e^{\hat{\kappa }}$.

This variable transformation necessitates a slight modification in the gradient calculations, which must be scaled by $e^{\kappa ^{(n)}}$ to adhere to the chain rule. For instance, step 9 in Algorithm 1 is updated to

$$\begin{aligned} &  \kappa ^{(i+1)} = \Pi _{\Theta _{\kappa }} \left[ \kappa ^{(i)} + e^{\kappa ^{(i)}} \frac{\delta _{\text {PGA}}^{(i+1)}}{2m}\right. \\ &  \left. \quad \sum _{j=1}^{m} \left\{ \left( (K + 1) \times C \times e^{-\kappa ^{(i)}} \right) - \Vert W^{(i,j)} \Vert _{F}^2 \right\} \right] , \end{aligned}$$

where $\Theta _{\kappa } = \{\log (\zeta _{\text {w}}) : \zeta _{\text {w}} \in \Theta _{\zeta _{\text {w}}}\}$ represents the permissible range of $\kappa$ values taken logarithmically.

## Appendix D Gradient and Step Size Computation for SOUL and AGD

### D.1 Gradient of ridge multinomial logistic regression

In the ULA step of the SOUL method (see (9) and Algorithm 1) and the computation of ${\hat{\textbf{W}}}$ (see Section 3.5), we need to compute the gradient of the logarithm of the ridge multinomial logistic regression model, that is

$$\begin{aligned}&\nabla _{\textbf{w}} \log p (\textbf{Y} \mid \tilde{\varvec{\Gamma }}^{\prime }, \textbf{W}, \hat{\zeta }_{\text {w}} ) = \nabla _{\textbf{w}} \left[ \sum _{i=1}^N \sum _{l=1}^C y_{i l} \textbf{w}_l^T\left\langle \widetilde{\varvec{\gamma }}_i^{\prime }\right\rangle \right. \\&\quad \left. + \sum _{i=1}^N \Biggl \langle \log \left[ \sum _{l=1}^C \exp \left( \textbf{w}_l^T \widetilde{\varvec{\gamma }}_i^{\prime }\right) \right] \Biggr \rangle + \frac{1}{2} \hat{\zeta }_{\text {w}} \Vert \textbf{W} \Vert _{F}^2 \right] . \end{aligned}$$

This gradient is a well-known result in the literature (see, e.g., (Hastie et al. 2009, Ex. 4.4)). Since it involves an expectation that does not have a closed-form solution, we must compute a Monte Carlo estimate of this log-sum-exp expectation term beforehand. This process was previously explained in Section 3.3. The gradient is then given by

$$\begin{aligned} \nabla _{\textbf{w}}&\log p (\textbf{Y} \mid \tilde{\varvec{\Gamma }}^{\prime }, \textbf{W}, \hat{\zeta }_{\text {w}} ) \approx - \frac{1}{J} \sum _{j=1}^J \widetilde{\varvec{\Gamma }}^{\prime (j) \textsf{T}}\\ &\quad \left[ p \left( \textbf{Y} \mid \tilde{\varvec{\Gamma }}^{\prime (j)}, \textbf{W}, \hat{\zeta }_{\text {w}} \right) - \textbf{Y}\right] + \hat{\zeta }_{\text {w}} \textbf{W} \\ &\quad :=\overline{\nabla }_{\textbf{w}} \log p (\textbf{Y} \mid \tilde{\varvec{\Gamma }}^{\prime }, \textbf{W}, \hat{\zeta }_{\text {w}} ), \end{aligned}$$

where we have generated a collection of $\tilde{\varvec{\Gamma }}^{\prime (1)}, \ldots , \tilde{\varvec{\Gamma }}^{\prime (J)}$ samples from $p ( \widetilde{\gamma }_{ik}=1 \mid \textbf{Y}, \textbf{T}, \widetilde{\varvec{\theta }}, \textbf{W} )$ to compute the Monte Carlo estimate, as explained in Section 3.3. In addition, $p (\textbf{Y} \mid \tilde{\varvec{\Gamma }}^{\prime }, \textbf{W}, \hat{\zeta }_{\text {w}} )$ is defined in (5). We have empirically found that $T = 30$ is enough to reach a good accuracy level while maintaining a low computational cost.

### D.2 Step size $\delta _{\text {ULA}}$ & $\delta _{\text {AGD}}$

Determining suitable step sizes for the ULA step in the SOUL method (see (9) and Algorithm 1) and AGD algorithm (see Section 3.5) is essential for guaranteeing convergence and computational efficiency. The literature offers well-established guidance on selecting the step size $\delta _{\text {AGD}}$. Specifically, $\delta _{\text {AGD}} \le 1 / L_f$ where $L_f$ is the Lipschitz constant of $\nabla _{\textbf{w}} \log p (\textbf{Y} \mid \tilde{\varvec{\Gamma }}^{\prime }, \textbf{W}, \hat{\zeta }_{\text {w}} )$.

The value of $L_f$ can be obtained from the Hessian of the objective function. For multinomial logistic regression (Böhning 1992), $L_f$ is given by

$$\begin{aligned} L_f = \lambda _{\max } \left[ \frac{1}{2} \left( {\textbf {I}}_C - \textbf{1}_C \textbf{1}_C^T / C \right) \otimes \tilde{\varvec{\Gamma }}^{\prime T} \tilde{\varvec{\Gamma }}^{\prime } \right] + \zeta _{\text {w}}, \end{aligned}$$

where *C* is the number of classes of the multinomial logistic regression model. This ensures that the AGD update step remains within a stable region, facilitating steady progress towards the optimal solution.

For the ULA in the SOUL method, when $\nabla _{\textbf{w}} \log p (\textbf{Y} \mid \tilde{\varvec{\Gamma }}^{\prime }, \textbf{W}, \hat{\zeta }_{\text {w}} )$ is Lipschitz continuous with Lipschitz constant $L_f$, it has been shown that $\delta _{\text {ULA}} \in (0, 2 / L_f]$ ensures that the Markov chain $(W^{(k)})_{k \ge 0}$ described in (9) is ergodic with stationary distribution close to the true target distribution (Durmus and Moulines 2017). Therefore, following both AGD and ULA specifications, we have decided to set $\delta _{\text {AGD}} = \delta _{\text {ULA}} = 0.95 / L_f$.

## Appendix E Simulation Study: Clustering Error

To complement the evaluation based on consensus scores presented in Section 4.1, we also assessed the accuracy of the recovered biclusters using the *Clustering Error* (CE) metric (Horta and Campello 2014; Nicholls and Wallace 2021). This metric quantifies the similarity between predicted and true biclusters based on the overlap of matrix elements, and is commonly used in benchmarking biclustering algorithms when ground truth is available.

Let $A_1, \ldots , A_p$ be the true biclusters and $B_1, \ldots , B_q$ the predicted biclusters. The clustering error is defined as:

$$\begin{aligned} \text {CE} = \frac{d_{\max }}{|U|}, \end{aligned}$$

where $d_{\max }$ is the total number of matrix elements correctly matched across an optimal pairing of biclusters, and |*U*| is the size of the union of all matrix elements involved in either the predicted or true biclusters.

Figure 11 shows the clustering similarity scores across 50 replicates for each method, including the FABIA and BicMix algorithms. As with the consensus score results in Section 4.1, the informative OG-SSLB implementation outperforms SSLB, FABIA and BicMix across all prior settings (BB, PY, IBP).

## Footnotes

1 Throughout, this superscript and subscript notation is utilised to denote the respective column and row vector of a matrix.

2 When denoting sequences of values, we use superscripts in parentheses instead of subscript and superscript notation to avoid confusion with matrix notation. For example, $\textbf{M}^{(i)}$ represents the $i$-th value in a sequence, rather than a power or column/row vector.
